# Decreased miR-451a in cerebrospinal fluid, a marker for both cognitive impairment and depressive symptoms in Alzheimer's disease

**DOI:** 10.7150/thno.81826

**Published:** 2023-05-15

**Authors:** Hu Feng, Panpan Hu, Yan Chen, Huaiqing Sun, Jiachen Cai, Xiaoxin He, Qiuchen Cao, Mengmei Yin, Yanli Zhang, Qian Li, Junying Gao, Charles Marshall, Chengyu Sheng, Jingping Shi, Ming Xiao

**Affiliations:** 1 Jiangsu Key Laboratory of Neurodegeneration, Nanjing Medical University, Nanjing, 211166, China.; 2 Brain Institute, Nanjing Brain Hospital, Nanjing Medical University, Nanjing, 210029, China.; 3 Department of Anesthetic Pharmacology, Faculty of Anesthesiology, Naval Medical University, Shanghai, 200082, China.; 4 Department of Neurology, the First Affiliated Hospital of Nanjing Medical University, Nanjing, 210029, China.; 5 Alice Lloyd College, Pippa Passes, KY, USA.; 6 Department of Neurology, the Affiliated Nanjing Brain Hospital of Nanjing Medical University, Nanjing, 210029, China.

**Keywords:** Alzheimer's disease, miR-451a, BACE1, depression, medial prefrontal cortex

## Abstract

**Background:** Alzheimer's disease (AD) patients are often accompanied by depressive symptoms, but its underlying mechanism remains unclear. The present study aimed to explore the potential role of microRNAs in the comorbidity of AD and depression.

**Methods:** The miRNAs associated with AD and depression were screened from databases and literature and then confirmed in the cerebrospinal fluid (CSF) of AD patients and different ages of transgenic APP/PS1 mice. AAV9-miR-451a-GFP was injected into the medial prefrontal cortex (mPFC) of APP/PS1 mice at seven months, and four weeks later, a series of behavioral and pathological analyses were performed.

**Results:** AD patients had low CSF levels of miR-451a, which was positively correlated with the cognitive assessment score, but negatively with their depression scale. In the mPFC of APP/PS1 transgenic mice, the miR-451a levels also decreased significantly in the neurons and microglia. Specific virus vector-induced overexpression of miR-451a in the mPFC of APP/PS1 mice ameliorated AD-related behavior deficits and pathologies, including long-term memory defects, depression-like phenotype, β-amyloid load, and neuroinflammation. Mechanistically, miR-451a decreased the expression of neuronal β-secretase 1 of neurons through inhibiting Toll-like receptor 4/Inhibitor of kappa B Kinase β/ Nuclear factor kappa-B signaling pathway and microglial activation by inhibiting activation of NOD-like receptor protein 3, respectively.

**Conclusion:** This finding highlighted miR-451a as a potential target for diagnosing and treating AD, especially for those with coexisting symptoms of depression.

## Introduction

Alzheimer's disease (AD) is the most prevalent neurodegenerative disease in the elderly, with a rising rate worldwide 1. Progressive cognitive decline is the typical clinical manifestation of AD, often accompanied by anxiety, depression, and other emotional disorders 2. Especially in a considerable proportion of AD patients, depression symptoms emerge before cognitive impairments 3-6. Therefore, exploring the common mechanisms of cognitive dysfunction and depressive behavior would benefit the early diagnosis and treatment of AD.

Epigenetics plays an essential role in the pathogenesis of multiple diseases 7. MicroRNAs (miRNAs) are a group of small noncoding RNAs that silence targeted genes at the post-transcriptional level 8. Accumulated literature has shown the involvement of miRNAs in various neurological disorders, including AD 9 and depression 10. Several miRNAs, such as miR-124, miR-135a, miR-132, miR-27a, and let-7, have been confirmed to be dysregulated in the brain tissue or serum of AD patients; and some of them are implicated in the accumulation of β-amyloid (Aβ) in AD transgenic mouse models 9, 11-14. Notably, the serum levels of miR-132, miR-27a, let-7, miR-124, and miR-135a are also changed in patients with depression 15-19. However, it remains to be elucidated which miRNAs are involved in AD patients coexisting with depression. Exploring this issue may benefit the early diagnosis and intervention of AD.

In the present study, we first screened for miRNAs associated with AD and depression from databases and literature. We identified an abnormal downregulation of miR-451a in the cerebrospinal fluid (CSF) of AD patients, and its levels correlated with cognition and depression scores. Consistently, miR-451a was also decreased in the medial prefrontal cortex (mPFC) of APP/PS1 transgenic mice. We further investigated whether overexpression of miR-451a in the mPFC rescued cognitive defects and depression-like symptoms of APP/PS1 mice. Finally, we addressed the potential mechanisms of miR-451a in regulating the pathogenesis of AD coexisting with depression.

## Materials and Methods

### Human samples

Human CSF samples were acquired from 47 participants, including 30 AD patients (48-76 years old) and 17 healthy volunteers (43-82 years old) who were recruited in the Affiliated Nanjing Brain Hospital of Nanjing Medical University (NMU) from March of 2019 to January of 2023 (Table S1-3). The recruitment of the AD patients was performed based on the current diagnostic criteria provided by the National Institute on Aging and the Alzheimer's Association Working Group (NIA-AA) in 2018. The guidelines are characterized by episodic memory loss in AD pathology supported by the evidence of CSF and imaging biomarkers 20. Each CSF sample (1 mL) was collected with BD vacutainer tubes and stored at -80 ℃ till further processing. The Montreal Cognitive Assessment (MoCA) is a rapid screening tool for cognitive impairments in attention and concentration, executive functioning, memory, language, visuospatial skills, abstract thinking, and computational and orienting skills. The total MoCA score is 30. The individual who acquires MoCA score < 26 is diagnosed as having cognitive impairment 21. The Hamilton Depression Scale (HAMD) is the most commonly used scale for clinically assessing depressive states. The scale is administered to patients by two trained raters in a combined HAMD examination, usually by talking and observing. A total score of less than 7 is rated as normal, and greater than 7 is assessed as the presence of depression 22. The recruitment, specimen collection, clinical assessment, and testing protocols of all participants were approved by the Ethics Committee of Nanjing Brain Hospital Affiliated with NMU (Ethics number: 2019-KY033-02).

### Animals

The male APP695/PS1-dE9 transgenic (APP/PS1) mice and wild-type (WT) littermates on a C57BL/6J background were purchased from Jackson Laboratories. All these mice were bred in the Experimental Animal Central of Nanjing Medical University. The mice were housed under a 12-h light/dark cycle in a temperature- and humidity-controlled environment with free access to food and water. Animal experiments were approved by the Institutional Animal Care and Use Committee of NMU (Approval No. IACUC-1812054).

### Virus construction and stereotactic injection

Viral constructs with adeno-associated virus serotype 9 (AAV9) vectors encoded Ad_OE-miR-451a blending pri-mmu-miR-451a, and GFP protein (Genechem, China) was designed to induce overexpression of miR-451a, and Ad_OE-scramble with only GFP protein as a negative control virus controlled by the CMV promoter. Mice were anesthetized by intraperitoneal (i.p.) injection of pentobarbital sodium (40 mg/kg) and placed in a stereotaxic apparatus. A total volume of 0.25 µL of AAV-miR-451a-OE (2 × 10^13^ viral particles per mL) or control virus was injected into the bilateral mPFC (anteroposterior: + 1.90 mm, mediolateral: ± 0.5 mm, dorsoventral: - 2.3 mm relative to bregma) by use of 1 μL Hamilton microsyringe in an 8-min period. The infusion needles were left in place for 5 min to limit virus backflow. After the scalp was sutured, the mice were kept on a heating pad to recover until fully awake. Then, the mice were raised for four weeks, followed by behavioral tests.

### Behavioral tests

#### Open field test

The open-field test assessed anxiety-like behavior and exploratory activity 23. The open field consisted of a plastic square blue box (60 × 60 × 25 cm) with an evident outlined center area (30 × 30 cm). Each mouse was placed in the middle of the box and allowed to explore the entire box freely for 5 min. The time spent in the central area and the number of central area entries during the test were recorded and analyzed.

#### Elevated plus maze test

Anxiety-like behavior was evaluated by the elevated plus maze test 24. The apparatus consisted of an elevated (100 cm above the floor) plus-shaped platform with four intersecting arms (50 × 10 cm each): two opposing open arms and two closed arms with 15 cm high walls. The four arms were extended from a central platform (10 × 10 cm). Each mouse was placed in the center square facing the open arm and left to explore for 5 min freely. The time duration and entries in the open arm were calculated and analyzed.

#### Novel object recognition test

The novel object recognition test assessed short-term recognition memory in rodents 25. This experiment used a rectangular box made of opaque plastic (50 × 35 cm) as the apparatus. Each mouse was placed in and allowed to habituate the apparatus for 5 min. Two identical plastic objects were placed on either corner of the box and 5 cm away from each adjacent arena wall. Each mouse was then placed in the arena and allowed to explore the arena and objects for 5 min. Two hours later, the mouse was again positioned in the same arena with one of the objects replaced with a novel object of a different shape and allowed to explore for another 5 min. A mouse was considered to be exploring an object when it was sniffing or touching it, and the time spent was measured. T1 represented the exploration time for the old object and T2 for the new object. The discrimination index was calculated as (T2-T1)/(T1+T2).

#### Y-maze test

Spatial memory was evaluated by the Y-maze test based on the nature of animals' spontaneous preference for spatial novelty 26. The experiment consisted of two 5-min phases with a 2-hour interval. In the first phase, the novel arm was blocked by a black baffle, and mice were allowed to move freely only in the other two arms. The novel arm was opened in the second phase, and the mouse could freely move through all three arms. The percentage of time spent and number of entries in the novel arm were calculated.

#### Three-chamber test

Social ability was evaluated by the three-chamber test 27. This test included the social preference test and social memory test. Mice were tested for social behavior in a chamber divided into three equal compartments (40 × 40 × 30 cm) by two square openings (8 × 8 cm) that allowed the test mice to enter. Before the social preference test, the mice were placed in the middle compartment and allowed to habituate for 5 min. In the social preference test, an unfamiliar male and age-matched mouse with the same background (stranger-1) was introduced into a mesh cage of one compartment. The testing mouse was then placed into the middle compartment and allowed to explore freely in the three compartments for 5 min. After a 10-min interval, the social memory test was conducted. Another age-matched stranger mouse (stranger-2) was introduced into the previously empty mesh cage. The testing mouse was again placed in the middle compartment and allowed to freely explore all three chambers for another 5 min. The percentage of time spent in each compartment was recorded.

#### Sucrose preference test

The sucrose preference test was operated to estimate the mouse anhedonia 28. The mice were trained to drink from two drinking bottles and then were deprived of drinking water but had free to acquire food for 24 h before the test. Each mouse was housed in a single cage and given one pot of normal water and the other pot of 1% sucrose solution for 12 h. At the end of the first half of the testing period, the positions of two pots were switched to avoid side preference. Consumption of sucrose solution or water was estimated by weighing the bottles before and after the test. The sucrose preference was calculated as follows: Sucrose preference rate (%) = sucrose consumption/total solution consumption × 100%.

#### Forced swimming test

The forced swimming test was designed to evaluate depression-like behavior. The mice were exposed to an inescapable stressful environment (water), and their immobile time was recorded to assess their despairing behavior 29. During the test, the mice were placed individually in a cylindrical glass cylinder (30 cm high, 10 cm in diameter) filled with 20 cm of water (24 °C). The duration of immobility of each mouse was analyzed during the last 4 min of the 5-min test.

#### Morris water maze

The Morris water maze was performed as previously described 30. Briefly, markers of different shapes were pasted around the swimming tank, and the water was made opaque with milk at a temperature of 22 °C. The mice were trained to find the hidden platform that submerged 1 cm beneath the water surface four times per day for six consecutive days. Once on the platform, the mice were allowed to remain on it for 15 s. If mice did not find the platform within 1 min, they were guided to the platform and allowed to stay for 15 s. On the seventh day, the hidden platform was removed; mice were placed in the quadrant opposite the target quadrant (previous location of the hidden platform) and allowed to swim for 1 min. The latency to reach the place of the platform, the percent time spent in the target quadrant, the crossing times to the platform regions, the swimming velocity, and the distance were recorded.

A digital video camera (Beijing Sunny Instruments Co. Ltd, China) connected to a computer was used to capture the activities of mice during the behavioral tests. The order of the behavioral tests was open field test (D30), elevated plus maze test (D31), novel objection recognition test (D32), Y-maze test (D33), three-chamber test (D34), sucrose preference test (D35), forced swimming test (D36) and Morris water maze (D37-D43). During the testing period, except for one dim light, all the lights in the room were turned off to ensure the comfort of the mice. At the end of each test, the mice returned to their home cages and rested for 24 hours. The experimental equipment was cleaned with 75% alcohol between each testing session to reduce cues that could affect trace and behavior. Two independent experimenters performed all tests, each blind to the treatment scheme.

### Cell culture and labeling

Murine neuronal lines Neuro-2a (N2a) and human embryonic kidney cells (HEK293) cells were bought from the American Type Culture Collection (ATCC). Cells cultured in the Dulbecco's Modified Eagle's medium (DMEM) that was supplemented with 10% fetal bovine serum (FBS, Gibco Thermo Fisher, USA), 100 U/mL of penicillin and 100 μg/mL of streptomycin (Invitrogen, USA) at 37 ℃ with 5% CO_2_.

Primary neuron cultures were prepared as previously described 31, the mPFC tissue was dissected out from postnatal day-1 WT and APP/PS1 mice and dissociated in DMEM containing dissociation buffer with 20-30 U/mL Papain (Sangon Biotech, China) and 2500 U DNase I (Sigma-Aldrich, USA) at 37 °C for 15 min, followed by FBS addition and 40 μm mesh filter to obtain dissociated cells. The suspension was centrifuged for 5 min at 1000 g and resuspended in neurobasal medium (Gibco) supplemented with 2% B27 (Gibco), 0.5 mmol/L glutamine (Sigma-Aldrich), and 1% penicillin/streptomycin (Gibco). Then, the cells were seeded into a poly-D-lysine (Sigma-Aldrich) precoated 6-well plates at a density of 5-10×10^6^ cells/mL and cultured at 37 °C and 5% CO_2_ for 24 h, then entirely replaced with culture medium. Finally, the culture medium was conducted a half-change every two days.

For primary microglial cultures 32, 1-day-old WT and APP/PS1 mice were decapitated, the mPFC was chopped with a 5 mL serological pipette, and the homogenates were obtained from spinning at 1000 g for 5 min. The supernatant was discarded, and the pellet was resuspended in a 10 mL pipette and drained through a 75 μm filter. The mPFC tissue was cultured in 75 cm^2^ flasks in 15 mL of culture medium that contained DMEM with 10% FBS, 100 U/mL penicillin, and 100 μg/mL streptomycin at 37 °C with 5% CO_2_. After 24 h, the new medium was added. The cultures were grown for an additional 10-14 days, and then the microglia were shaken from the astrocyte feeder layer for 2 h, resuspended in a culture medium, and plated.

For miR-451a knockdown, miR-451a inhibitors (RiboBio, Guangzhou, China) were used, and double-stranded miR-451a designed as mimic (RiboBio) to overexpress miR-451a. For toll-like receptor 4 (TLR4) downregulation, a double-stranded siRNA targeting mouse TLR4 or scrambled negative control (Shanghai GenePharma Co. Ltd, China) was used. For dual luciferase assays, the GV272 vector (GeneChem Biotechnology, Shanghai, China) was used, and the WT or mutation (Mut) 3′ UTR sequences of TLR4 are listed in Table S4. In short, N2a, HEK293, primary neurons, and primary microglial cells were plated in 12-well plates at 2 × 10^5^ cells per well. After 24 h hatching, miR-451a mimic (50 nM), inhibitor (100 nM), or negative control (50 nM) were transfected with Lipofectamine 2000 (Invitrogen). The TLR4 siRNA (100 nM) and overexpression plasmid were transfected into cells according to the manufacturer's instructions. Cellular RNA, proteins, and fluorescence were obtained 48 h after transfection.

### Immunofluorescence and fluorescence in situ hybridization (FISH)

Frozen brain sections were incubated with blocking buffer (5% bovine serum albumin, 0.3% PBS-Triton X-100) for 1 h at room temperature and then incubated with appropriate dilutions of primary antibodies (Table S5) overnight at 4 °C. After being washed with PBS for 3 times, brain sections were incubated with secondary immunofluorescence antibodies for 2 h at room temperature. After washed, brain sections were incubated with 1:1000 DAPI (4′, 6-diamidino-2-phenylindole) (Invitrogen; Cat. #D21490) diluted by PBS for 5 min at room temperature. The sections were then washed with PBS and covered with glass coverslips. The images were captured using a confocal microscope (Zeiss LSM710, Germany).

The locked nucleic acid-modified miR-451a probes labeled with Cy3 were designed and constructed (Shanghai GenePharma Co. Ltd), and the probe sequence was AAC+TCAGTAA+TGGTAACGGT+TT, "+" indicated the modification site of locked nucleotide. The probe signals were detected with a fluorescent in situ hybridization kit (RiboBio). In short, after pre-hybridization brain sections were incubated with miR-451a probes in hybridization solution at 37 °C overnight. The sections were washed with PBS and performed the immunofluorescent procedure described above. Two brain sections containing the mPFC or hippocampus were averaged for each mouse, and four to six mice were averaged for each group.

### RNA extraction and analysis

The total RNA was isolated from human CSF, the mouse serum, mPFC, and hippocampus using RNAiso Plus (TAKARA, Japan) according to the manufacturer′s protocols and quantified by Nanodrop 2000 (Thermo, USA). The detection of miRNA and mRNA was carried out in 96-well plates. Quantitative real-time PCR (qRT-PCR) was performed using an ABI 7300 Fast Real-Time PCR System (Applied Biosystems, USA).

Reverse transcription PCR for miRNAs was carried out using a Poly (A) tailing reaction and universal cDNA synthesis kit (Sangon Biotech Co., Ltd. Shanghai, China). Then the qRT-PCR was conducted using SYBR Green MicroRNAs qPCR Kit (B532461, Sangon Biotech, Shanghai, China). The small nucleolar RNA (snoRNA) RNU6B was used as endogenous controls for miRNA.

The mRNA was reverse transcripted using HiScript III RT SuperMix for qPCR (+gDNA wiper) (R323-01, Vazyme Biotech Co., Ltd, USA), and mRNA expression was assessed using a Taq Pro Universal SYBR qPCR Master Mix (Vazyme) by real-time PCR. Results were analyzed, presented relative to threshold cycle (CT) values, and then converted to fold changes. Glyceraldehyde-3-phosphate dehydrogenase (GAPDH) mRNA was used as an endogenous control. PCR was repeated at least twice for two separately prepared sets of samples. The expression of miRNAs and mRNAs was presented as fold change relative to control. The primer information is listed in Table S6.

### Western blotting

For Western blot analyses, the homogenized protein samples of the brain and cells were loaded onto 8-15% Tris/tricine SDS gels and electrophoretically transferred to PVDF membranes (Millipore, USA). After blocking for 1 h in 5% nonfat milk/TBST, the membranes were incubated at 4 °C overnight with primary antibodies (Table S5). The membranes were then incubated with horseradish peroxidase-conjugated secondary antibodies (1:2000; Vector Laboratories, USA) for 1 h at room temperature. Blots were visualized using an ECL kit (Tanon, Shanghai, China). Westerns blots were quantified using ImageJ software (NIH, USA) using an internal GAPDH loading control for each blot analyzed. Fold changes in expression were calculated relative to the control group. The experiments were repeated at least twice for each sample, and six mice in each group were used for statistical analysis.

### Enzyme-linked immunosorbent assay (ELISA)

For ELISA of interleukin-1β (IL-1β), interleukin-6 (IL-6), tumor necrosis factor-α (TNF-α), soluble Aβ_1-40_, and Aβ_1-42_ concentrations, mPFC tissues and primary neuron cells were homogenized in lysis buffer with protease inhibitors. After mixed, samples were centrifuged at 12,000 g for 20 min, and the supernatant was measured with ELISA kits (Aβ_1-40_ and Aβ_1-42_ purchased from Jingmei Bio, Shenzhen, China; IL-1β, IL-6, and TNF-α purchased from ExCell Bio, Shanghai, China) according to the manufacturer′s protocols. All samples were tested at least twice.

### β-site APP cleaving enzyme 1 (BACE1) activity analysis

BACE1 enzyme activity detection was performed according to the manufacturer′s instructions (β-Secretase Activity Fluorometric Assay Kit, Sigma-Aldrich). Briefly, the lysis buffer of the primary cells was centrifuged at 10,000 g for 5 min, and a portion was used for the quantitative protein experiment with the BCA method. Then 50 μL of cell lysis was added, followed by the addition of reaction buffer and substrate. Fluorescence for each sample was measured using the full-wavelength microplate reader. The analyses were repeated at least twice in all samples.

### Statistical analysis

Statistical tests for each figure were justified to be appropriate using Prism 8.0 (GraphPad Software, Inc.). Data are presented as mean ± SEM. Two-tailed unpaired student's *t*-tests were utilized for comparisons between two groups, and one-way ANOVA was used to compare three independent groups. Multifactorial comparisons were analyzed by two-way ANOVA with Tukey's post-hoc test. Pearson's correlation analysis was conducted to evaluate the correlation between CSF miR-451a levels and indicators of AD patients. Significance was determined as* p* values < 0.05.

## Results

### CSF miR-451a levels in AD patients were positively correlated with MoCA score and negatively correlated with HAMD score

**Figure 1:**
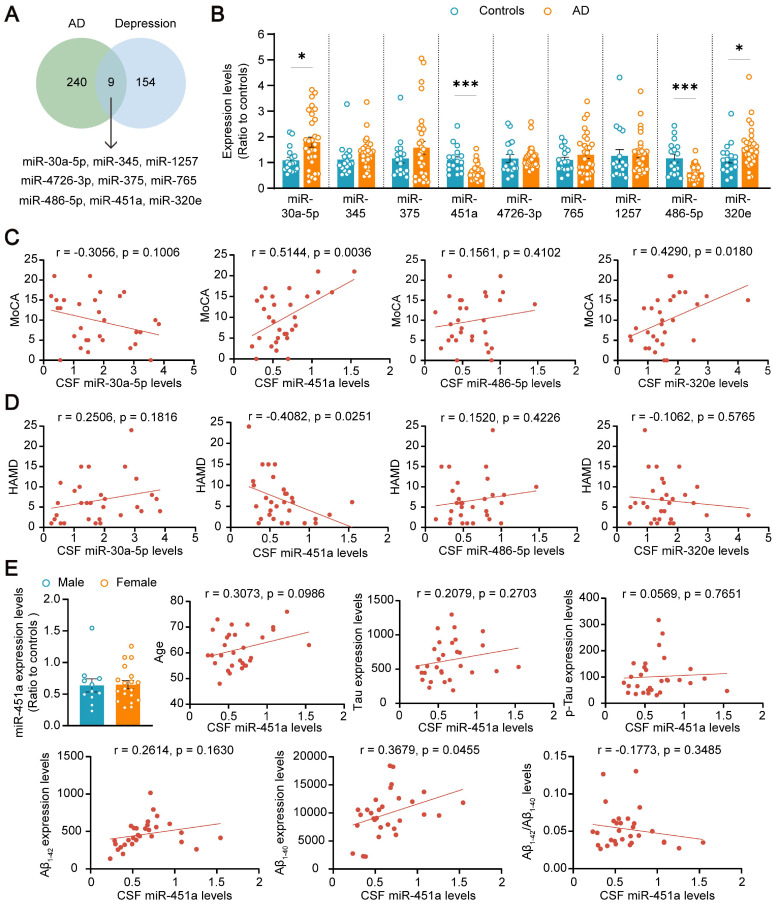
**MiR-451a is altered in AD patients. (A)** The results of database search and screening. Nine miRNAs were screened out by multiple literature and GEO databases of AD and Depression. **(B)** qRT-PCR evaluating the levels of miR-30a-5p, miR-345, miR-375, miR-451a, miR-4726-3p, miR-765, miR-1257, miR-486-5p, and miR-320e in the CSF of AD patients and control individuals. **(C)** Correlation analysis between the relative CSF levels of miR-30a-5p, miR-451a, miR-486-5p, and miR-320e in AD patients and the total score of MoCA, respectively. **(D)** Correlation analysis between the relative CSF levels of each miRNA mentioned above in AD patients and the total score of HAMD.** (E)** Analysis of the relationship between CSF miR-451a levels and gender, age, Aβ levels, and Tau levels, respectively. Data are presented as mean ± SEM. n = 30 for AD patients (11 males and 19 females) and n = 17 for controls (11 males and 6 females). Significance was evaluated with Student's *t*-test (**B, E**) or Pearson's correlation test (**C, D, E**). *p < 0.05, ***p < 0.001.

To explore the potential role of miRNAs in the comorbidity of AD and depression, both AD and depression-related miRNAs were screened from the literature 33-35, database GSE157239 of AD, and GSE81152 and GSE58105 of depression. Nine miRNAs including miR-30a-5p, miR-345, miR-375, miR-451a, miR-4726-3p, miR-765, miR-1257, miR-486-5p, and miR-320e were successfully screened out (Figure 1A). The expression levels of these nine miRNAs in the CSF samples of AD patients and healthy volunteers were further validated by qRT-PCR. Only four miRNAs, including miR-30a-5p, miR-486-5p, miR-320e, and miR-451a, changed in AD patients, compared with healthy controls (Figure 1B). The correlation between the four miRNAs and cognitive score or depression severity in AD patients was further analyzed. The results showed that the miR-451a levels were positively correlated with the MoCA score but negatively with the HAMD score (Figure 1C, D). Furthermore, the CSF levels of miR-451a were lower in AD patients with depressive symptoms than in AD patients without depression (Figure S1A). The miR-320e levels were positively correlated with the MoCA score (Figure 1C), and the miR-1257 levels were negatively correlated with the HAMD score (Figure S2B). Otherwise, the associations between the other six miRNA levels and MoCA or HAMD were not observed (Figure S2A, B). After exclusion of confounding factors including gender and age in four changed miRNAs, the miR-451a levels were positively correlated with Aβ_1-40_ levels (Figure 1E), and the miR-30a-5p levels were negatively correlated with total Tau levels in the CSF (Figure S3A). Moreover, we did not observe any associations between miR-486-5p, miR-320e levels and indicators of AD patients including age, gender, Aβ levels (Aβ_1-42_, Aβ_1-40_, and Aβ_1-42_/Aβ_1-40_), and Tau levels (total Tau and p-Tau) (Figure S3B, C). We also analyzed miR-451a levels of Early-Onset AD (EOAD, age < 65) and Late-Onset AD (LOAD, age ≥ 65) and did not find significance between the two groups (Figure S1B). Based on this, we focused on exploring the potential involvement of miR-451a in the co-pathogenesis of AD and depression using APP/PS1 double transgenic mouse model for AD.

### Decreased miR-451a levels in the CSF and mPFC of APP/PS1 mice

**Figure 2:**
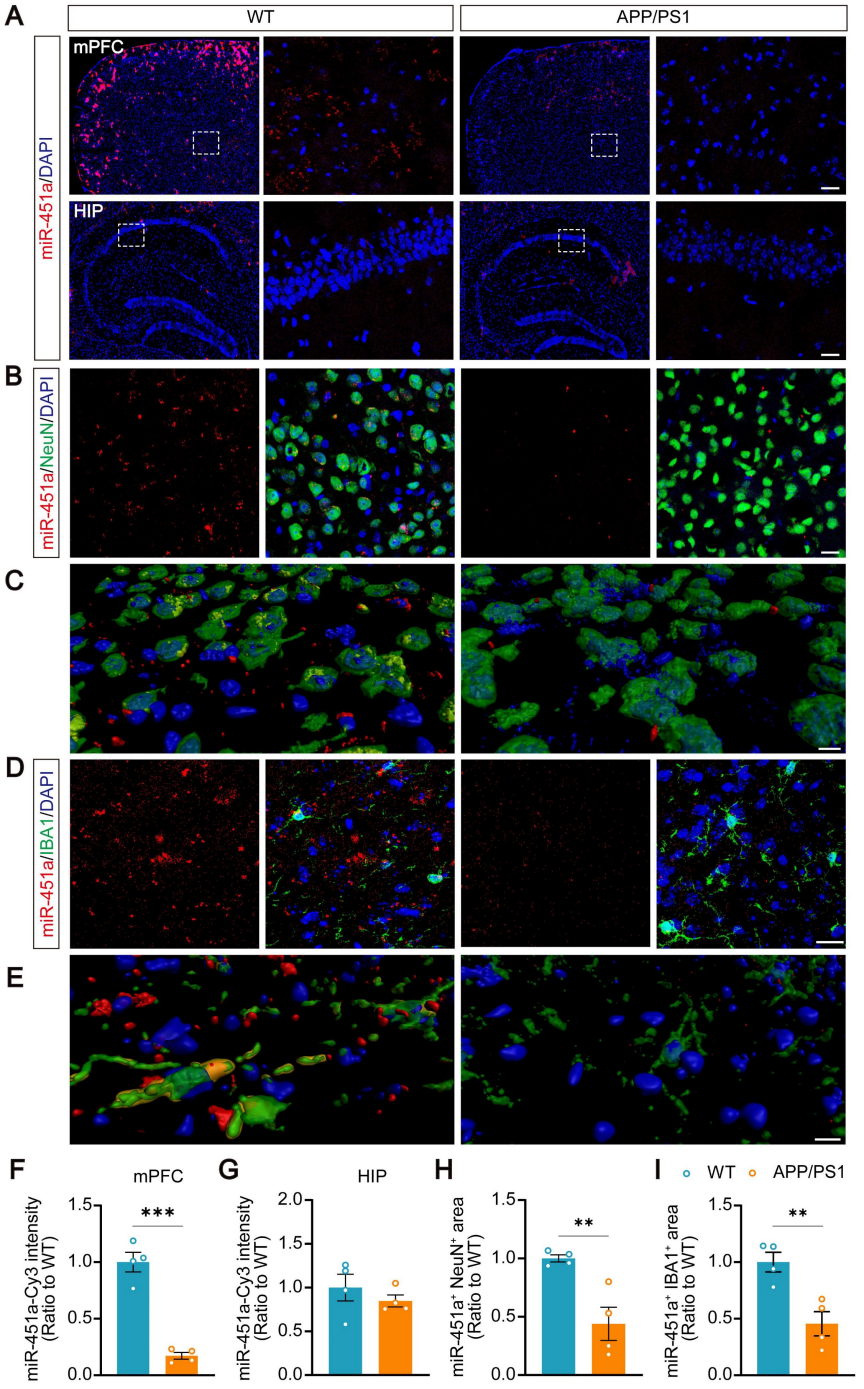
** Decreased expression of miR-451a in the mPFC neurons and microglia in APP/PS1 mice. (A)** Representative RNA FISH images showed decreased miR-451a expression in the mPFC (upper panel) but not in the hippocampus (lower panel) of 7-month-old APP/PS1 mice compared to age-matched WT mice. Scale bar, 20 µm.** (B)** Representative imaging showed that miR-451a was partially colocalized within NeuN positive neurons and down-regulated expression in APP/PS1 mice, compared with WT mice. Scale bar, 20 μm. **(C)** Three-dimensional reconstruction and surface rendering for FISH of miR-451a within neurons. Red showed outside cells, and yellow showed inside cells. Scale bar, 10 μm. **(D)** A small proportion of miR-451a was also expressed in the IBA1-positive microglia and significantly down-regulated in APP/PS1 mice. Scale bar, 20 μm. **(E)** Three-dimensional reconstruction and surface rendering for FISH of miR-451a in microglia. Red showed outside cells, and yellow showed inside cells. Scale bar, 10 μm. **(F)** The fluorescence intensity of miR-451a in the mPFC. **(G)** The fluorescence intensity of miR-451a in the hippocampus (HIP). **(H)** The relative area percentage of miR-451a and NeuN double-positive signals in the mPFC. **(I)** The relative area percentage of miR-451a and IBA1 double-positive signals in the mPFC. Data are presented as mean ± SEM. n = 4 per group. Significance was evaluated with Student's *t*-test. **p < 0.01, ***p < 0.001.

Serum miR-451a levels in APP/PS1 transgenic mice were examined at ages 3, 5, 7, and 9 months and decreased from 7 months compared to age-matched controls (Figure S4A). Previous studies have reported that miR-451a is enriched in the brain and participates in glioma cell invasiveness 36, neuroinflammation, oxidative stress 37, and neuronal apoptosis 38. The dynamic changes of miR-451a in the hippocampus and mPFC, two brain regions markedly impacted in AD or depression, were investigated 39-41. Also, at 7 and 9 months old, the levels of miR-451a decreased significantly in the mPFC of APP/PS1 mice (Figure S4B). However, its expression in the hippocampus was similar between APP/PS1 and WT mice in the above age groups (Figure S4C). Decreased miR-451a levels were also observed in the CSF of 7-month-old APP/PS1 mice (Figure S4D, E). The fluorescence in situ hybridization confirmed a decrease in miR-451a expression in the mPFC of the 7-month-old APP/PS1 mice (Figure 2A, F). Double staining revealed that miR-451a was localized in both NeuN positive neurons (Figure 2B, C) and ionized calcium-binding adapter molecule 1 (IBA1) positive microglia (Figure 2D, E) in the mPFC, which dramatically reduced in APP/PS1 mice (Figure 2H, I). The levels of miR-451a were extremely low in the hippocampus, and there was no significant difference between the two genotypes of mice (Figure 2A, G).

### Overexpression of miR-451a in the mPFC alleviated long-term spatial memory and depression-like behavior in APP/PS1 mice

**Figure 3:**
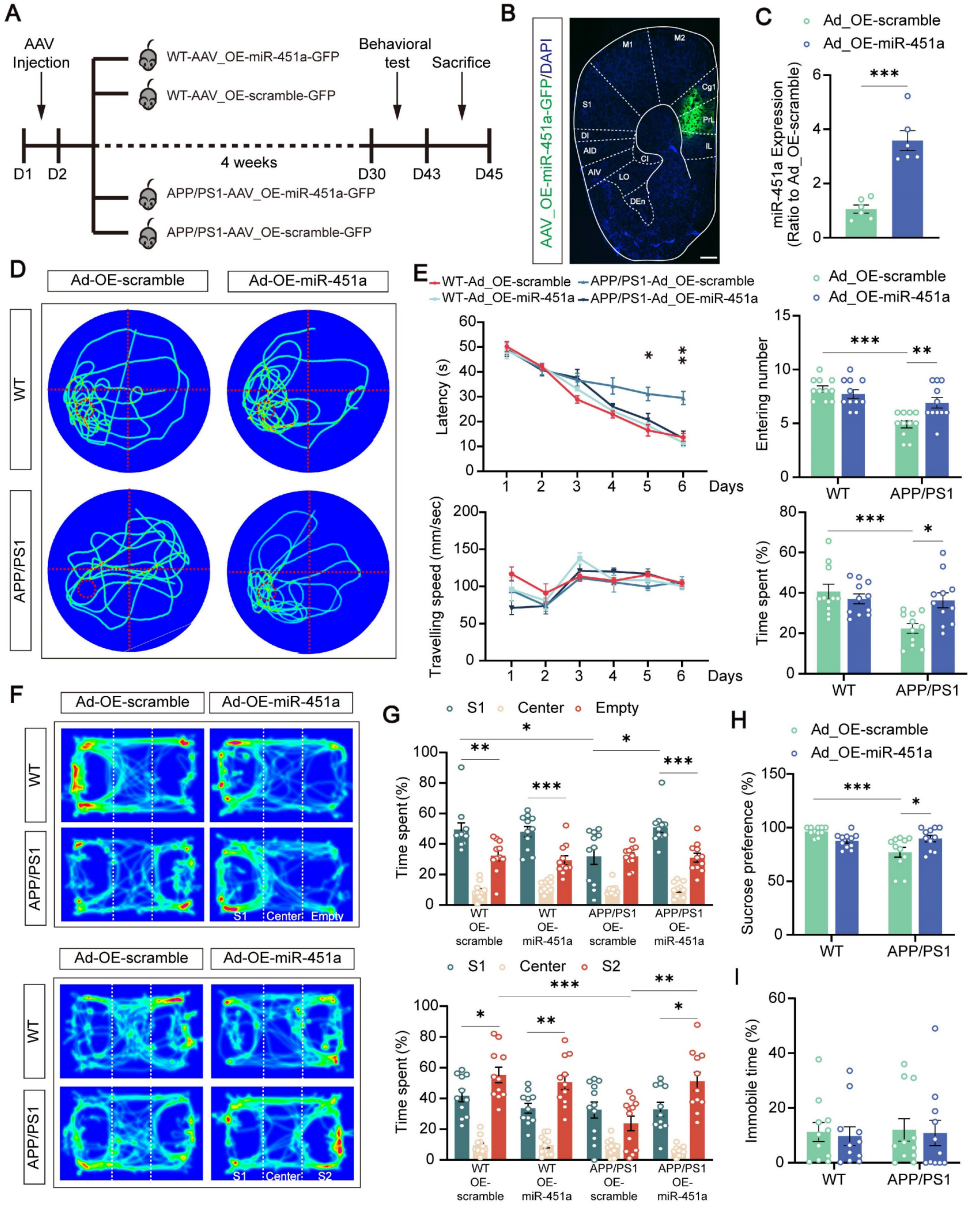
** Overexpression of miR-451a rescued behavior alterations in APP/PS1 mice. (A)** Schematic diagram illustrating experimental time course for APP/PS1 and WT mice that underwent AAV injection, behavioral tests, and sacrifices (OE-miR-451a for miR-451a overexpression and OE-scramble for a negative control). **(B)** The representative fluorescence image of the AAV virus-infected slice. Scale bar, 400 μm. IL: infralimbic cortex; PrL: prelimbic cortex; Cg1: cingulate cortex, area 1; M2: secondary motor cortex; M1: primary motor cortex; S1: primary somatosensory cortex; DI: dysgranular insular cortex; AID: agranular insular cortex, dorsal part; AIV: agranular insular cortex, ventral part; CI: caudal interstitial; LO: lateral orbital cortex; DEn: dorsal endopiriform nucleus. **(C)** Injection of AAV_OE-miR-451a-GFP increased miR-451a level in the mPFC of APP/PS1 mice, as revealed by qRT-PCR results. **(D)** Traces on the probe trial on day 7 in the Morris water maze. **(E)** The latencies to the target quadrant containing platforms, swimming speed, entering time, and time spent in the target quadrant in the Morris water maze. **(F)** Track of mice in the three-chamber sociability test, including social preference test and social memory test. **(G)** The percentage of interaction time spent in three chambers in the social preference test (S1: Stranger mice 1) and social memory test (S2: Stranger mice 2). **(H)** Quantification of sucrose intake preference in the sucrose preference test. **(I)** Quantification of immobility time in the forced swimming test. Data are presented as mean ± SEM. n = 6 per group for **(C)** and n = 11 per group for (**E, G, H, I**). Significance was evaluated with Student's *t*-test (**C**), two-way ANOVA with Tukey post- hoc test (**E, G, H, I**), repeated two-way measures ANOVA with Tukey post-hoc test (latency and speed in **E**), or one-way ANOVA with Tukey post-hoc test (comparison within group in **G**). *p < 0.05, **p < 0.01, ***p < 0.001.

Previous studies, including those from our group, demonstrated that apart from age-dependent cognitive defects, APP/PS1 mice also exhibit depression-like behavior 42, 43. To investigate whether miR-451a was involved in these abnormal behaviors, AAV9-miR-451a-GFP or control virus (AAV9-scramble-GFP) were injected into the mPFC of APP/PS1 mice at 7 months to induce and measure the miR-451a overexpression of WT and APP/PS1 mice. Four weeks later, a series of behavioral tests were performed (Figure 3 A, B). As expected, AAV9-miR-451a-GFP injection in the mPFC of APP/PS1 mice upregulated miR-451a gene levels by 3-4 folds compared with the AAV9-scramble-GFP control group, as shown by qRT-PCR (Figure 3C).

In the Morris test for long-term spatial memory, such virus-induced miR-451a overexpressed APP/PS1 mice showed a significantly shortened latency to find the hidden platform during the training phase (Figure 3D) and crossed the target quadrant more often and spent more time in the quadrant in which the platform was located during the probe test compared with the AAV9-scramble-GFP injected control group (Figure 3E). However, such improved memory benefits by miR-451a overexpression failed to be observed during the Y maze and novel objection recognition short-term memory test (Figure S5A-D). In addition, miR-451a upregulation partially rescued the decreased sucrose preference (Figure 3H) and social preference and social memory in the three-chamber test (Figure 3F, G), without affecting immobility time in the forced swimming test (Figure 3I) or anxiety-like performance in the open field and elevated plus maze of the APP/PS1 mice (Figure S5E-H). Taken together, these results demonstrated that overexpression of miR-451a in the mPFC specifically alleviated the impaired long-term spatial memory and depressive-like behavior of APP/PS1 mice.

### Overexpression of miR-451a ameliorated AD neuropathology in the mPFC of APP/PS1 mice

**Figure 4:**
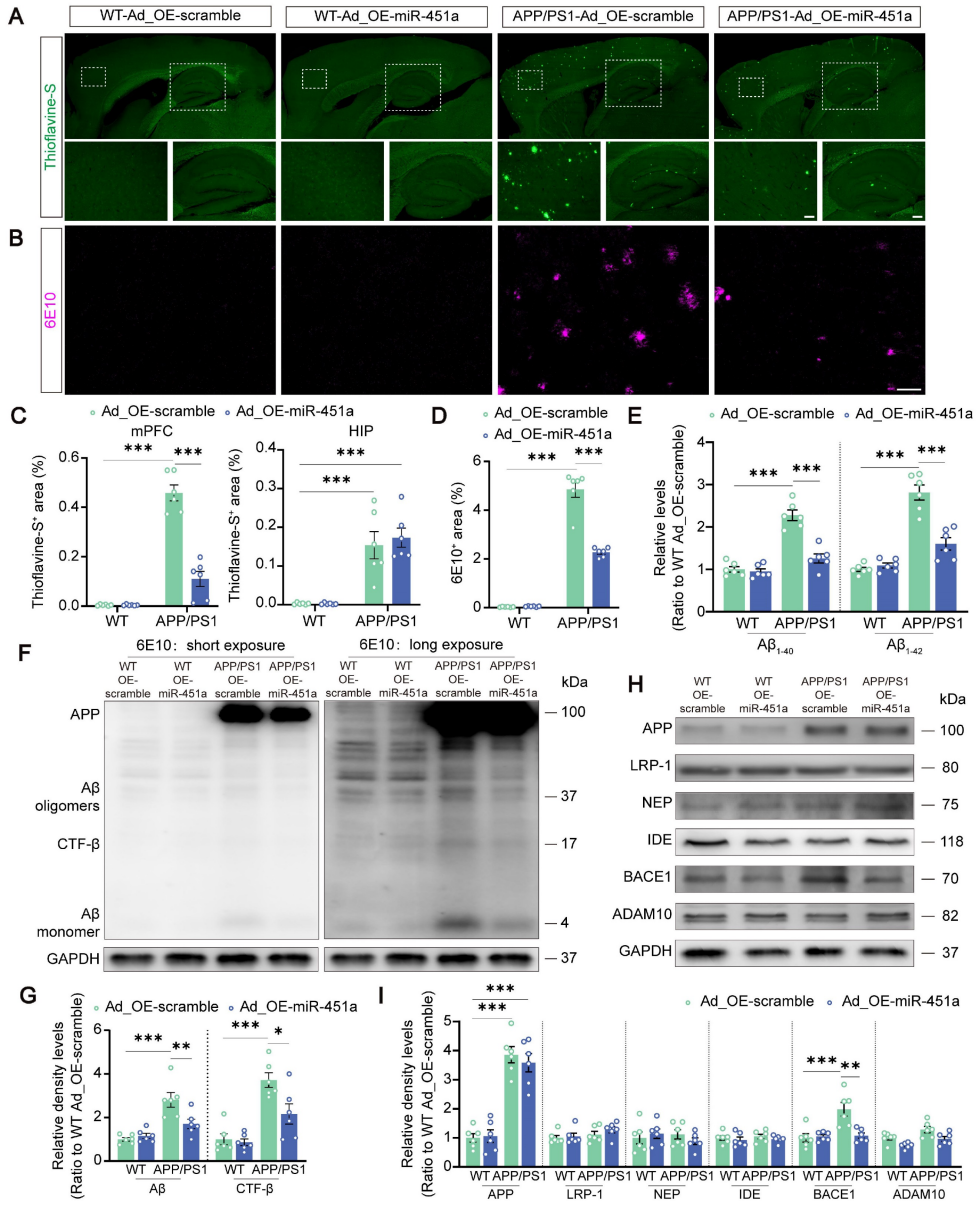
** Overexpression of miR-451a ameliorated Aβ load and inhibited BACE1 expression in the mPFC of APP/PS1 mice. (A)** Representative images of thioflavine-S^+^ plaques in the mPFC and hippocampus of each group. Scale bar, 20 μm for the mPFC and 50 μm for the hippocampus (HIP), respectively. **(B)** Representative image of 6E10^+^ plaques in the mPFC of each group. Scale bar, 50 μm.** (C)** The percentage of thioflavine-S^+^ area in the mPFC and hippocampus, respectively. **(D)** The percentage of 6E10^+^ area in the mPFC. **(E)** ELISA analyses of soluble Aβ_1-40_ levels and Aβ_1-42_ levels in the mPFC of each group. **(F, G)** Representative Western blot bands and densitometry analysis for Aβ and CTF-β in the mPFC of each group.** (H, I)** Representative Western blot bands and densitometry analysis for APP, LRP-1, NEP, IDE, ADAM10, and BACE1 in the mPFC of each group. Data are presented as mean ± SEM. n = 6 per group. Significance was evaluated with two-way ANOVA with Tukey post-hoc test. *p < 0.05, **p < 0.01, ***p < 0.001.

To investigate the involvement of miR-451a in AD-like pathology of APP/PS1 mice, Aβ metabolism, plaque deposition, and glial activation in the mPFC and hippocampus were examined. Our results demonstrated that AAV9-miR-451a-GFP treatment significantly reduced thioflavine-S-positive Aβ plaques in the mPFC rather than hippocampus of APP/PS1 mice (Figure 4A, C). Consistently, immunofluorescence of 6E10-positive plaques also confirmed the result (Figure 4B, D). Moreover, overexpression of miR-451a decreased soluble Aβ_1-40_ and Aβ_1-42_ levels in the mPFC of APP/PS1 mice (Figure 4E). In addition, miR-451a overexpression successfully rescued pathologically increased β-secretase metabolites of APP, including Aβ monomer, oligomers, and β-C-terminal fragment (CTF-β) and its key regulator, beta APP cleaving enzyme 1 (BACE1) in the mPFC of such AD model mice (Figure 4F-I). However, no other regulators were affected by miR-451a overexpression (Figure 4H, I). Further, immunofluorescence confirmed a down-regulated BACE1 expression in the mPFC rather than hippocampus of miR-451a overexpressed APP/PS1 mice (Figure S6A-F). Our results proposed that miR-451a overexpression in the mPFC of APP/PS1 mice could alleviate the AD pathology of APP/PS1 mice by specifically targeting BACE1 to counteract the deleterious β-secretase metabolites of APP.

### MiR-451a down-regulated BACE1 through targeting TLR4/IKKβ/NF-κB signal pathway

**Figure 5:**
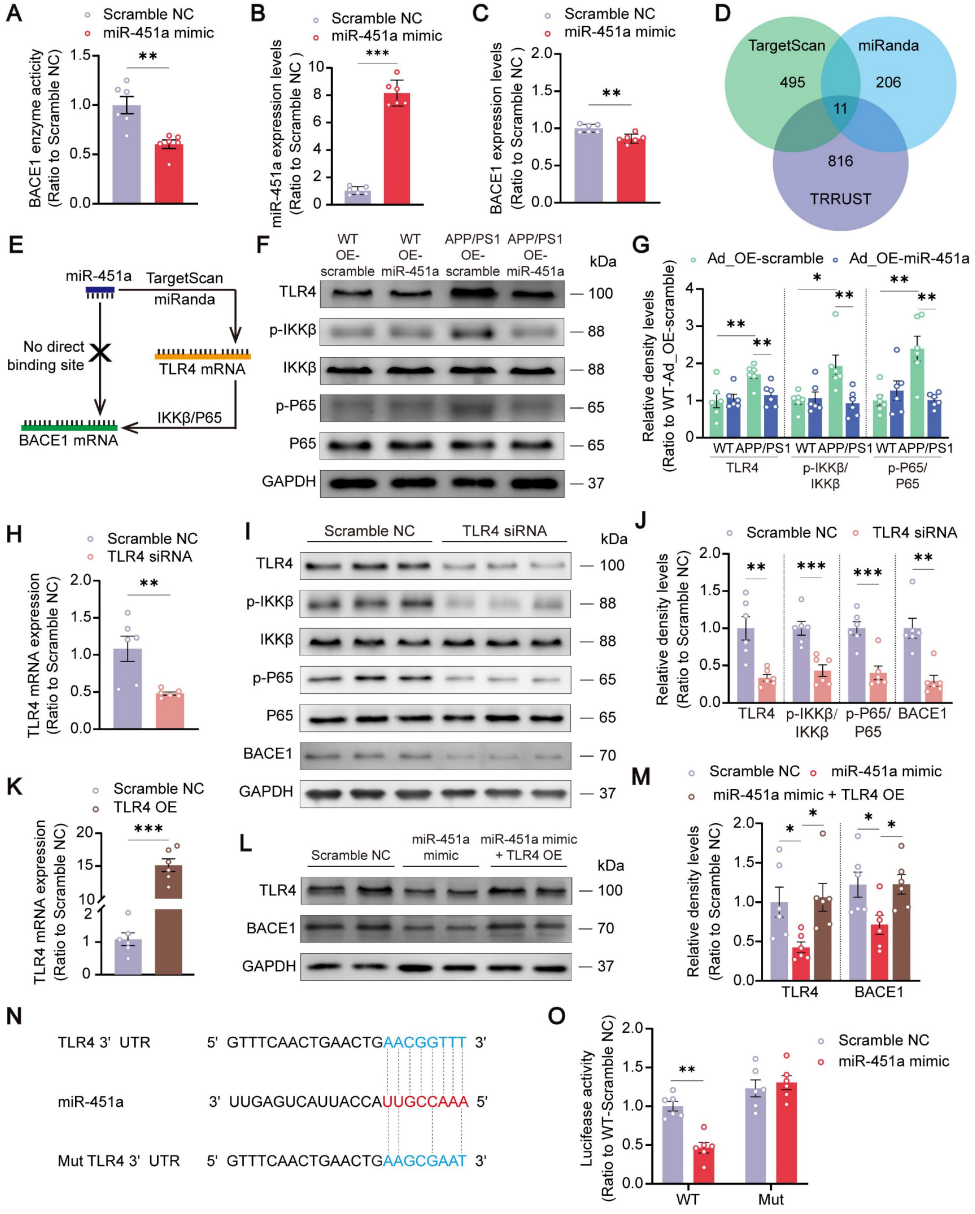
** MiR-451a downregulated BACE1 transcription by targeting TLR4/IKKβ/NF-κB signal pathway. (A)** The activity of BACE1 enzyme in primary cortical neurons after miR-451a mimic treatment.** (B)** qRT-PCR showed that miR-451a expression was significantly up-regulated in 293T cells after miR-451a mimic (50 nM) treatment. **(C)** qRT-PCR showed that BACE1 mRNA levels were prominently decreased in 293T cells after miR-451a mimic (50 nM) treatment.** (D)** A Venn diagram showing transcription factors (816 genes) from TRRUST overlapped with the target genes of miR-451a predicted by miRanda (206 genes) or TargetScan (495 genes). **(E)** The direct targets of miR-451a predicted by TargetScan and miRanda did not include BACE1 but include TLR4, which mediated IKKβ/NF-κB pathway to downregulate BACE1 production. **(F, G)** Representative Western blot bands and densitometry analysis for TLR4/IKKβ/NF-κB pathway-related proteins showed that overexpression of miR-451a reduced TLR4 expression, p-IKKβ/IKKβ and p-P65/P65 in the mPFC of APP/PS1 mice.** (H)** qRT-PCR showed that TLR4 siRNA (100 nM) reduced the mRNA levels of TLR4 in N2a cells. **(I, J)** Representative Western blot bands and densitometry analysis showed that TLR4 siRNA (100 nM) reduced the levels of TLR4, p-IKKβ/IKKβ, p-NF-κB/NF-κB and BACE1 in N2a cells.** (K)** qRT-PCR showed that overexpression of TLR4 increased the mRNA levels of TLR4 in N2a cells. **(L, M)** Representative Western blot bands and densitometry analysis showed that miR-451a mimic reduced the protein levels of TLR4 and BACE1, and overexpression of TLR4 normalized the levels of these proteins in N2a cells. **(N)** The sequence analysis indicated the nucleotide base pairing of the miR-451a binding region in TLR4 3′UTR. The mutant sequence of TLR4 3′UTR for luciferase analysis was provided at the bottom. **(O)** The quantification of luciferase reporter assays of WT and Mut sequence of TLR4 3′UTR in 293T cell after treatment of miR-451a mimic. Data are presented as mean ± SEM. n = 6 per group. Significance was evaluated with Student's *t*-test (**A, B, C, H, J, K**), two-way ANOVA with Tukey post-hoc test (**G, O**), or one-way ANOVA with Tukey post-hoc test (**M**). *p < 0.05, **p < 0.01, ***p < 0.001.

It is well-known that miRNAs regulate gene expression by inhibiting the gene's translation or enhancing the degradation of their target mRNAs 44. Therefore, in the next *in vitro* experiments, the miR-451a mimic was used to determine how miR-451a regulated BACE1 expression. The transfection of miR-451a mimic induced a significant increase of miR-451a and a substantial decrease of BACE1 mRNA expression in 293T cells (Figure 5B, C). In primary cortical neurons, miR-451a mimic also successfully decreased activity of the BACE1 enzyme (Figure 5A), which further confirmed the regulation of BACE1 by miR-451a.

However, the results analyzed by the Targetscan and miRanda databases showed that BACE1 was not a direct downstream target of miR-451a (Figure 5E). Therefore, 11 potential downstream targets of miR-451a, including transcription factor 2 (ATF2), Friend leukemia integration 1 (FLI1), myelocytomatosis oncogene (MYC), nuclear factor I/B (NFIB), peroxisome proliferative activated receptor gamma co-activator 1 alpha (PPARGC1A), sine oculis homeobox 1 (Six1), T-box 19 (Tbx19), toll-like receptor 4 (TLR4), WW domain containing transcription regulator 1 (WWTR1), Y box protein 1 (YBX1), and zinc finger protein of the cerebellum 3 (Zic3) were screened out by TRRUST and analyzed (Figure 5D). Among the 11 candidates, only FLI1, TLR4, and WWTR1 showed increased mRNA levels in the mPFC of APP/PS1 mice (Figure S7A). After miR-451a overexpression, only the TLR4 level was normalized in this AD model mice (Figure S7B). Further investigation via bioinformatics analysis of the GSE33000 dataset of mPFC in AD patients downloaded from the GEO database supported the forementioned findings, showing that the expression level of TLR4 was increased in the mPFC of AD patients (Figure S7C). Thus, miR-451a may target BACE1 by directly regulating the TLR4.

It has been shown that NF-κB/P65 could specifically interact with their binding element in the BACE1 promoter regions 45. In agreement with this, we found that overexpression of miR-451a decreased the activation of TLR4/IKKβ/NF-κB signal pathway in the mPFC of APP/PS1 mice (Figure 5F, G). To further explore whether miR-451a could regulate BACE1 expression by inactivating TLR4/IKKβ/NF-κB pathway, TLR4 siRNA was used to block the translation of TLR4 in the N2a cells. As expected, TLR4 siRNA markedly reduced mRNA levels of TLR4 (Figure 5H) and protein levels of TLR4, p-IKKβ, p-NF-κB, and BACE1 in N2a cells (Figure 5I, J). Then, TLR4 overexpression plasmid-treated N2a cells showed a significantly elevated mRNA level of TLR4 (Figure 5K) and counteracted miR-451a mimic-induced protein decline of TLR4 and BACE1 (Figure 5L, M).

Moreover, the miR-451a inhibitor increased the activation of TLR4/IKKβ/NF-κB signal pathway in N2a cells (Figure S7D, E). To demonstrate the direct regulation of TLR4 by miR-451a, we constructed the WT 3′UTR region of TLR4, which contains the miR-451a binding site, and a 3′UTR mutation with a variant at the miR-451a binding site. Then, we subcloned them in the GV272 vectors and transfected them into 293T cells with either the miR-451a mimic or scrambled control. The WT and Mut 3′UTR sequences of TLR4 were listed in Table S4. We found that the miR-451a mimic suppressed the luciferase intensity in cells transfected with the WT construct but not the mutated construct (Figure 5N, O).

### MiR-451a alleviated neuroinflammation via inhibiting NLRP3 activation in microglia

**Figure 6:**
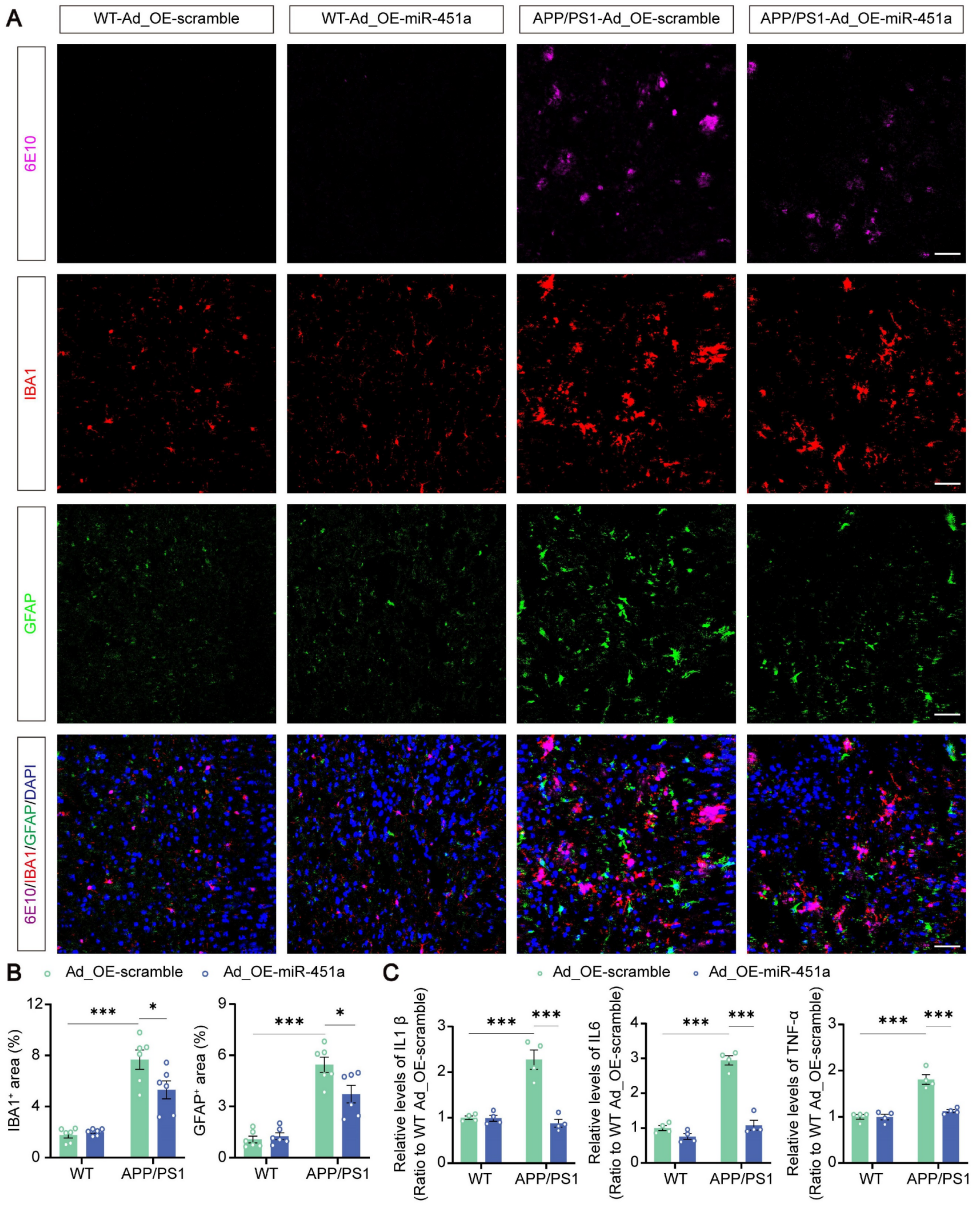
** MiR-451a inhibited neuroinflammation in the mPFC of APP/PS1 mice. (A)** Representative image of 6E10 (Magenta), IBA1 (Red), GFAP (Green), and DAPI (Blue) in the mPFC of each group. Scale bar, 50 μm. **(B)** The percentage of IBA1^+^ and GFAP^+^ area in the mPFC. **(C)** ELISA analyses of IL-6, IL-1β, and TNF-α levels in the mPFC of each group. Data are presented as mean ± SEM. n = 6 per group for (**B**) and n = 4 per group for (**C**). Significance was evaluated with two-way ANOVA with Tukey post-hoc test. *p <0.05, ***p < 0.001.

**Figure 7:**
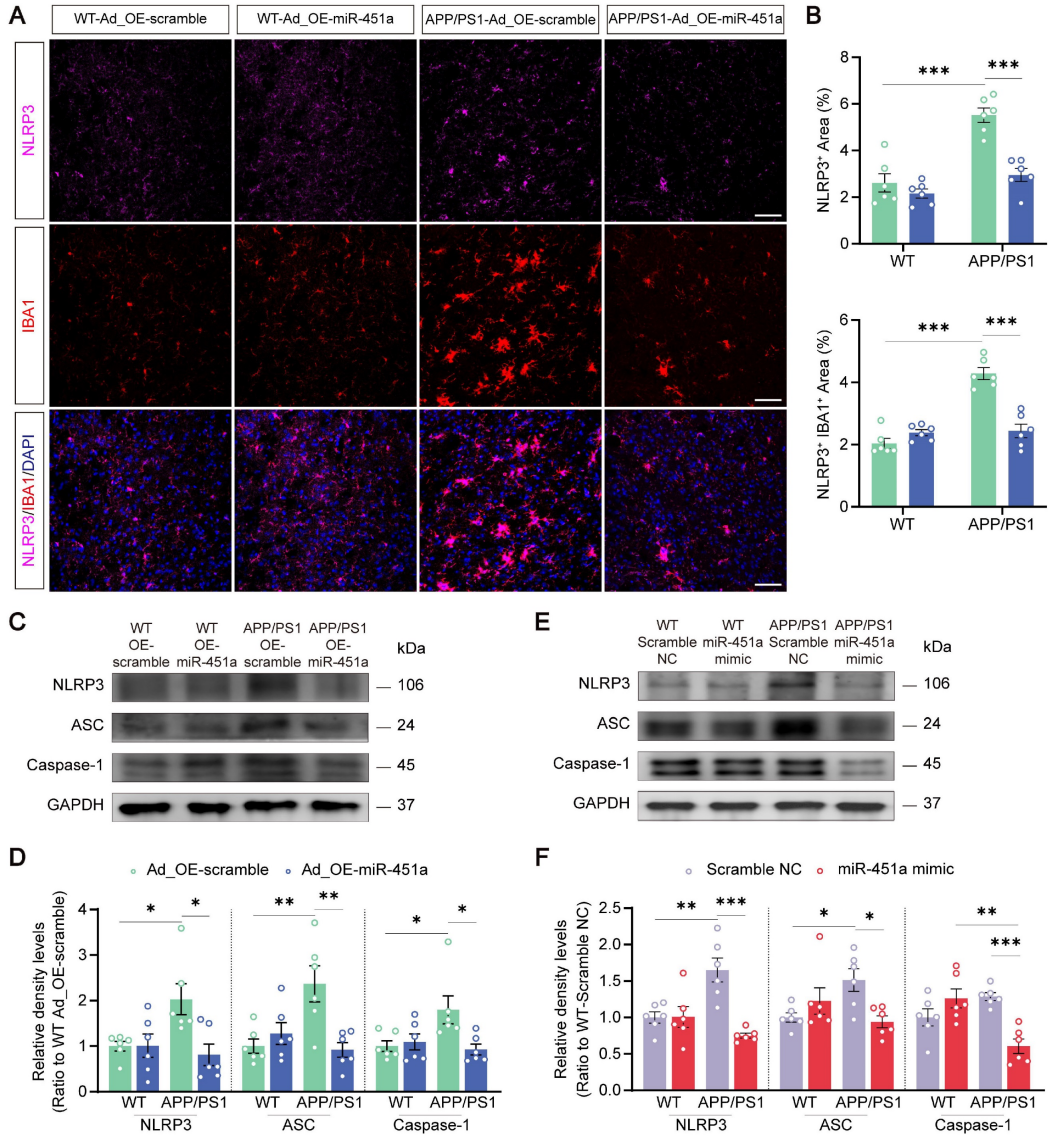
** MiR-451a regulated neuroinflammation in the microglia via NLRP3/ASC/Caspase-1 pathway. (A)** Representative image of NLRP3 (Magenta), IBA1 (Red), and DAPI (Blue) in the mPFC of each group. Scale bar, 50 μm.** (B)** The percentage of NLRP3^+^ and NLRP3^+^IBA1^+^ area in the mPFC.** (C, D)** Representative Western blot bands and densitometry analysis of NLRP3, ASC, and Caspase-1 in the mPFC of each group. **(E, F)** Representative Western blot bands and densitometry analysis of NLRP3, ASC, and Caspase-1 in the primary microglia after treatment of miR-451a mimic. Data are presented as mean ± SEM. n = 6 per group. Significance was evaluated with two-way ANOVA with Tukey post-hoc test. *p <0.05, **p < 0.01, ***p < 0.001.

Neuroinflammation is confirmed as a common pathogenic manifestation of AD and depression 46, 47, in which miRNAs play a non-negligible role 48. As mentioned above, miR-451a was also down-regulated in mPFC microglia of APP/PS1 mice (Figure 2D). Therefore, we examined its role in reactive gliosis and found that overexpression of miR-451a reduced activated microglia and astrocytes with decreasing of Aβ plaques in the mPFC of APP/PS1 mice, as revealed by a low percentage area of IBA1, GFAP and 6E10 immunostaining (Figure 6A, B), but no significant difference in the hippocampus (Figure S8A-D). Moreover, AAV9-miR-451a-GFP-induced miR-451a overexpressed APP/PS1 mice had low levels of inflammatory cytokines, including IL-1β, IL-6, and TNF-α (Figure 6C). Recently, it has been reported that miR-451a relieves chronic inflammatory pain by inhibiting microglia activation-mediated inflammation 49 and suppressing NLRP3-induced proinflammatory cascade signaling 50. Consistently, our results revealed that miR-451a overexpressed APP/PS1 mice had decreased NLRP3 expression on the microglia (Figure 7A, B) as well as its downstream effectors ASC and Caspase-1 in the mPFC (Figure 7C, D). In* vitro*, miR-451a mimic also down-regulated NLRP3/ASC/Caspase-1 signal pathway in the primary microglia from APP/PS1 mice (Figure 7E, F).

### MiR-451a protected primary neurons from APP/PS1 mice

**Figure 8:**
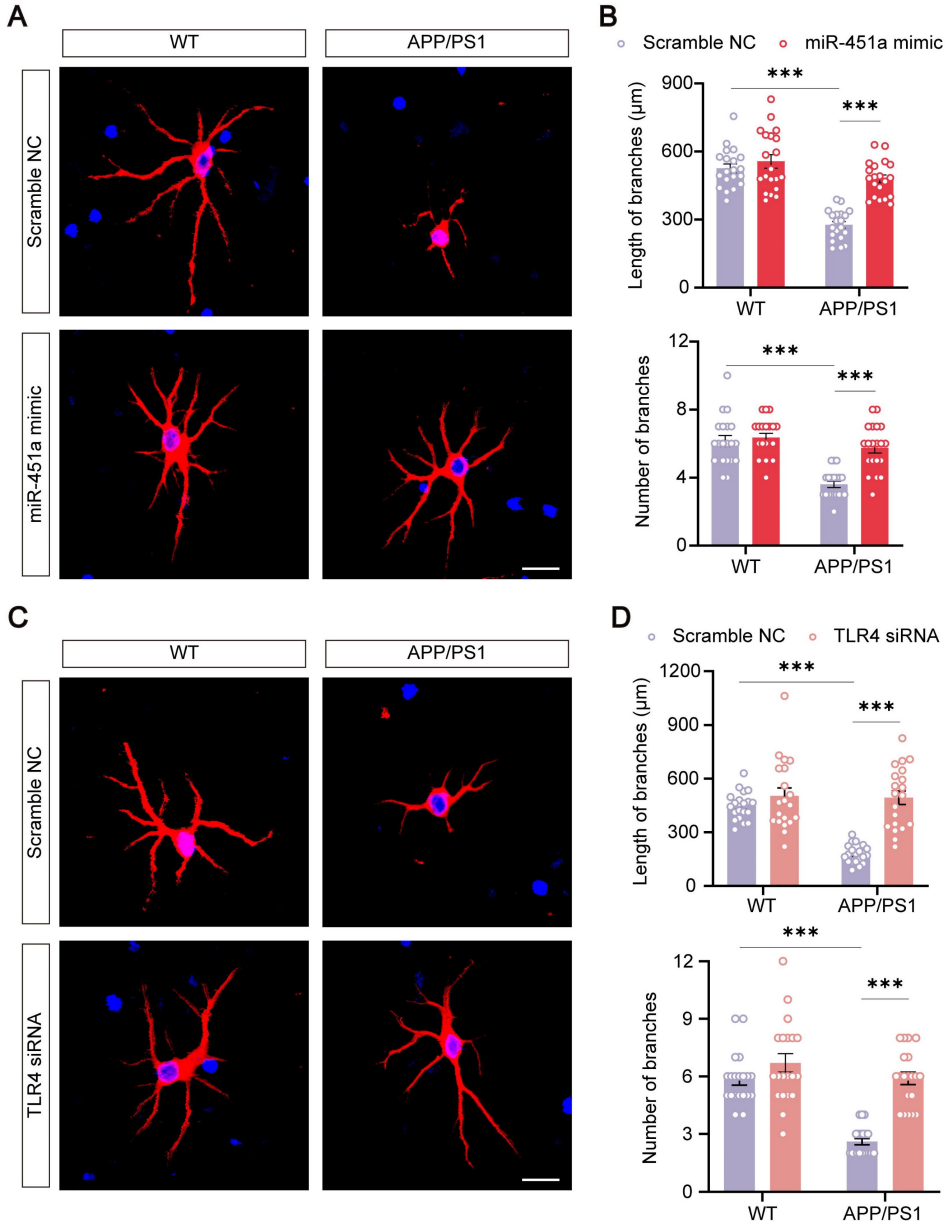
** MiR-451a protected against neuritic atrophy in primary neurons of APP/PS1 mice. (A)** Representative images of MAP2 of primary neurons treated with either miR-451a mimic (50 nM) or scramble NC. Scale bar, 20 μm. **(B)** Quantification of the neuritic length and branches number of primary neurons after miR-451a mimic treatment. **(C)** Representative images of MAP2 of primary neurons treated with either TLR4 siRNA (100 nM) or scramble NC. Scale bar, 20 μm. **(D)** Quantification of the neuritic length and branches number of primary neurons after TLR4 siRNA treatment. Data are presented as mean ± SEM. n = 20 per group. Significance was evaluated with two-way ANOVA with Tukey post-hoc test. ***p < 0.001.

Previous literature suggested that neuronal degeneration occurred in the cerebral cortex of APP/PS1 mice, and the neurons were prone to atrophy when cultured *in vitro* 51-54. Our results consistently showed that primary cortical neurons derived from newborn APP/PS1 mice had significantly decreased length and number of neuritic branches compared with those from WT mice. MiR-451a mimic remarkably rescued neuronal degeneration in culture (Figure 8A, B). In addition, miR-451a mimic significantly rescued synaptic protein loss of APP/PS1 mice in *vivo* and *vitro* (Figure S9A-D). Moreover, miR-451a mimic decreased Aβ_1-40_ and Aβ_1-42_ deposition in primary neurons from APP/PS1 mice. Supernatant centrifuged from primary neurons in APP/PS1 mice with the treatment of miR-451a mimic produced less Aβ_1-40_ rather than Aβ_1-42_ (Figure S10A, B). Our results also showed that BACE1 and downstream products, such as sAPPβ and CTF-β, increased in primary neurons from APP/PS1 mice and decreased with treatment of miR-451a mimic (Figure S10C, D). As discussed earlier in the previous section, miR-451a could modulate TLR4 to reduce the production of Aβ in the mPFC of APP/PS1 mice. Therefore, we explored whether the dendritic protective effect from miR-451a was caused by TLR4 inhibition. TLR4 mRNA levels were blocked by siRNA to examine BACE1 expression and Aβ production in the primary neurons. Our results showed that TLR4 siRNA caused a decrease in Aβ_1-42_ levels but failed to influence Aβ_1-40_ contents within primary neurons from APP/PS1 mice. Supernatant centrifuged from primary neuronal medium showed less Aβ_1-40_ and Aβ_1-42_ concentrations after treatment of TLR4 siRNA (Figure S10E, F). Moreover, we found that BACE1, sAPPβ, and CTF-β increased in primary neurons from APP/PS1 mice and decreased with the treatment of TLR4 siRNA (Figure S10G, H). Interestingly, we found that TLR4 siRNA rescued neuronal atrophy (Figure 8C, D). The above results demonstrated that TLR4 modulated by miR-451a produced a protective effect for primary neurons from APP/PS1 mice.

## Discussion

Clinical research shows that 50% of AD patients are accompanied by depression, which is also the leading risk factor in AD 55, 56. Therefore, it is necessary to explore the co-pathogenesis of AD and depression. As an essential component of epigenetics, miRNAs have attracted more attention in neuropsychiatric diseases, including AD 57, 58. In this study, we screened nine miRNAs involved in both AD and depression. Changes of these nine miRNAs in AD or/and depression according as previous literature were summarized in Table S7.

Consistent with the previous literature 59-61, we verified the changes of these nine miRNAs in the CSF of AD patients and found that miR-30a-5p and miR-320e levels increased. In contrast, miR-486-5p and miR-451a levels decreased compared to those in age-matched controls. However, few studies addressed the correlation between CSF miRNA levels and cognitive dysfunction or depressive behavior in AD cases. In this study, we conducted the correlation analysis and found that only CSF miR-451a levels were related to both MoCA and HAMD scales, indicating an involvement in the co-pathogenesis of AD and depression. Interestingly, we also found that CSF miR-451a levels were lower in depressed AD patients than in AD patients without depression, suggesting that miR-451a may be an essential regulatory molecule for depressive symptoms in AD.

MiR-451a is located on the long arm of chromosome 17 and is enriched in blood, the pancreas, and other tissues 62-64. MiR-451a is also expressed in brain tissue. We found that miR-451a was specifically expressed in the neurons and microglia, consistent with the previous reports 50, 65. Alterations of miR-451a are observed in systemic lupus erythematosus, rheumatoid arthritis, and other diseases 63, 66. In 2008, Cogswell et al. first reported that miR-451 decreased in the CSF of AD patients 33, which was confirmed by subsequent studies 67. In addition, the proportion of patients with EOAD in our study was high, reaching 60%. This is because the majority of elderly LOAD patients are reluctant to receive CSF puncture surgery mainly due to poor overall physical health status. We further confirmed the decrease in miR-451a CSF levels in patients with EOAD and LOAD and found no significant difference between the two types of AD, which is consistent with previous literature 60.

Although a large amount of literature reported dysregulation of miR-451a in patients with AD, its contribution to AD-related pathology remained elusive. Therefore, in this study, we characterized dynamic changes of miR-451a in the blood and CSF of APP/PS1 mice, an extensively used AD mouse model. We further demonstrated that miR-451a was down-regulated in both mPFC neurons and microglia of APP/PS1 mice from the age of 7 months. Still, the underlying mechanism of its down-regulation needs further exploration.

Recent studies have shown that RNA degradation is enhanced in the brain of AD patients 68. The stability of miRNAs in the brain depends largely on the two RNA-binding proteins, RBFOX and ZFP36. The imbalance of RBFOX1 has been shown to cause the instability of the miRNAs that regulate the gene expression of synaptic plasticity-related proteins, subsequently impairing synaptic function in AD 68. In addition, miR-451a is negatively regulated by DNMT3B in bladder cancer 69. DNMT3B, as a DNA methylation enzyme, increases the risk of AD by interacting with the APOE4 70 and is up-regulated in peripheral blood mononuclear cells in LOAD patients 71. Therefore, it is worth further studying whether RBFOX1 and/or DNMT3B affect brain miR-451a expression during AD.

Previous literature reported that 7-month-old APP/PS1 mice showed a significant increase in BACE1 expression in the mPFC 72, which is consistent with the current finding. Increased BACE1 expression promotes Aβ production, and secondary neuroinflammation, synapse and dendritic degeneration 72, 73. It has been reported that miR-451a is down regulated in glioma, and overexpression of miR-451a can target IKKβ via NF-κB signal pathway, thereby inhibiting the growth of glioma cells *in vitro* and *in vivo* 74. We found that miR-451a inhibited IKKβ/NF-κB signal pathway via decreasing TLR4 expression, subsequently down-regulating BACE1 and reducing Aβ production. In addition, high BACE1 level is also detected in the peripheral blood of patients with depression 75. It remains to be determined whether alterations of BACE1 expression are involved in the depression-like phenotype of AD mice.

In addition to neurons, previous literature suggested that miR-451a regulates the activation of microglia in animal models of chronic pain 49, spinal cord injury 50, and gliomas 76. Our results indicated that overexpression of miR-451a in APP/PS1 mice reduced the inflammatory response and NLRP3 activation. Overexpression of miRNA-451a also markedly reduced the level of NLRP3 in primary microglia. This suggests that NLRP3 is another target of miR-451a. This conclusion has been reconfirmed in a recent study that miR-451 directly targets NLRP3 and suppresses NLRP3-induced production of the inflammatory cytokines IL-1β and IL-18 in a mouse model of spinal cord injury 50.

It has been reported that the mPFC is involved in various behavioral activities. For example, our recent study reported that altered miR-124/Nr4a1 signal in the mPFC was involved in social behavioral defects of socially isolated mice 77. The mPFC pyramid neurons regulate the spatial working memory 78, and the initiation of triggering receptor expressed on myeloid cell-2 (TREM2) transcription by activation of nuclear factor erythroid 2-related factor 2 (Nrf2) increases the microglial M2 phenotype in the mPFC, thus ameliorating depression-like behavior 79. In the current study, we found that overexpression of miR-451a in the mPFC could improve long-term memory, social ability, and depression-like behavior in APP/PS1 mice. It is known that memory is initially stored in the hippocampus, but over time, memory will slowly consolidate in the cortex for permanent storage 80. In agreement with this view, overexpression of miRNA-451a in the mPFC only improves long-term memory defects without affecting the hippocampus-related short-term memory.

In conclusion, we identified that miRNA-451a was closely related to cognitive impairment and depression in AD patients. We further verified that miR-451a was involved in regulating long-term memory and depression-like phenotype in AD model mice. We also demonstrated that overexpression of miRNA-451a in the mPFC inhibited the production of BACE1 via TLR4/IKKβ/NF-κB pathway and the glial inflammatory response by down-regulating NLRP3 activation, thereby reducing the neuronal degeneration. These results have revealed miRNA-451a as a novel target for the prevention and treatment of AD, especially for those with coexisting symptoms of depression.

## Supplementary Material

Supplementary figures and tables.

## Abbreviations

AAV: Adeno-associated virus serotype
AD: Alzheimer's disease
ADAM10: A disintegrin and metalloprotease 10
AID: Agranular insular cortex, dorsal part
AIV: Agranular insular cortex, ventral part
APOE4: Apolipoprotein E 4
APP: Amyloid β precursor protein
ATF2: Activating transcription factor 2
BACE1: β-site APP cleaving enzyme 1
Cg1: Cingulate cortex, area 1
CI: Caudal interstitial
CSF: Cerebrospinal fluid
CT: Threshold cycle
CTF-β: β-C-terminal fragment
DAPI: 4′, 6-diamidino-2-phenylindole
DEn: Dorsal endopiriform nucleus
DI: Dysgranular insular cortex
DNMT3B: DNA Methyltransferase 3 Beta
ELISA: Enzyme-linked immunosorbent assay
EOAD: Early-Onset AD
FISH: Fluorescence in situ hybridization
FLI1: Friend leukemia integration 1
GAPDH: Glyceraldehyde-3-phosphate dehydrogenase
HAMD: Hamilton Depression Scale
HEK293: Human embryonic kidney cells
IBA1: Ionized calcium binding adapter molecule 1
IDE: Insulin-degrading enzyme
IKKβ: Inhibitor of kappa B Kinase β
IL: Infralimbic cortex
IL-1β: Interleukin-1β
IL-6: Interleukin-6
LO: Lateral orbital cortex
LOAD: Larly-Onset AD
LRP1: Low-density lipoprotein receptor related protein 1
M1: Primary motor cortex
M2: Secondary motor cortex
MAP2: Microtubule Associated Protein 2
miRNA: microRNA
MoCA: Montreal Cognitive Assessment
mPFC: Medial prefrontal cortex
MYC: Myelocytomatosis oncogene
NEP: Neutral endopeptidase
NFIB: Nuclear factor I/B
NF-κB: Nuclear factor kappa-B
NLRP3: NOD-like receptor protein 3
Nrf2: Nuclear factor erythroid 2-related factor 2
PPARGC1A: Peroxisome proliferative activated receptor gamma co-activator 1 alpha
PrL: Prelimbic cortex
PS1: Presenilin-1
PSD95: Postsynaptic density protein 95
qRT-PCR: Quantitative real-time PCR
RBFOX1: RNA Binding Fox-1 Homolog 1
S1: Primary somatosensory cortex
sAPPβ: Soluble amyloid precursor protein beta
SCI: Spinal cord injury
Six1: Sine oculis homeobox 1
SYP: Synaptophysin
Tbx19: T-box 19
TLR4: Toll like receptor 4
TNF-α: Tumor necrosis factor-α
TREM2: Triggering receptor expressed on myeloid cell-2
WWTR1: WW domain containing transcription regulator 1
YBX1: Y box protein 1
ZFP36: Zinc Finger Protein 36
Zic3: Zinc finger protein of the cerebellum 3
