# Supplementary material for: Decreased miR-451a in cerebrospinal fluid, a marker for both cognitive impairment and depressive symptoms in Alzheimer's disease
Source: Theranostics. 2023 May 15;13(9):3021–40. doi: 10.7150/thno.81826 (PMC10240826; doi:10.7150/thno.81826)
Supplement: Supplementary file 1 — Supplementary figures and tables. [file thnov13p3021s1.pdf]

Table S1: Cut-off values of AD biomarkers in CSF

Index	Reference range
$A\beta_{1-42}$	< 550 pg/ml suggests amyloid lesions
	551-650 pg/ml suggests a suspicious
	≥ 651 pg/ml suggests normal range
$A\beta_{1-40}$	≥ 7000 pg/ml suggests normal range
	< 7000 pg/ml suggests deposition
$A\beta_{1-42}/A\beta_{1-40}$	≤ 0.05 suggests positive
	> 0.05 suggests negative
t-Tau	≤ 399 pg/ml suggests normal range
	> 399 pg/ml suggests neuronal cell death
p-Tau	≤ 50 pg/ml suggests normal range
	> 50 pg/ml suggests neurofibrillary tangles

All indexes were tested by ELISA

Table S2: Information of AD patients

No.	Age	Gender	MoCA	HAMD	$A\beta_{1-42}$	$A\beta_{1-40}$	$A\beta_{1-42}$/ $A\beta_{1-40}$	t-Tau	p-Tau
AD1	63	Male	10	7	383.82	9976.34	0.04	793.66	108.52
AD2	71	Male	4	12	385.06	5729.66	0.07	469.49	50.28
AD3	76	Female	16	3	261.35	9542.1	0.03	470.67	94.21
AD4	59	Male	3	24	136.42	2751.04	0.05	530.17	78.11
AD5	66	Female	15	1	601.5	12004.44	0.05	453.15	88.47
AD6	62	Female	0	8	519.44	15085.24	0.03	190.6	40.91
AD7	69	Female	14	10	332.40	10561.27	0.03	344.29	65.79
AD8	63	Male	21	6	413.92	11817.66	0.04	531.35	46.87
AD9	59	Female	17	15	322.310	10566.66	0.03	311.75	65.33
AD10	55	Male	17	8	1015.44	18224.68	0.06	1112.11	85.31
AD11	55	Female	2	2	541.5	10550.32	0.05	449.36	37.73
AD12	66	Female	3	15	572.8	8984.91	0.06	528.35	39.95
AD13	56	Female	5	6	631.42	14496.16	0.04	884.85	221.96
AD14	60	Female	5	15	451.52	7425.3	0.06	707.64	58.64
AD15	56	Male	5	5	256.566	9663.57	0.03	445.73	100.98
AD16	54	Female	6	9	584.88	18405.4	0.03	1297.55	316.99
AD17	57	Male	6	1	538.54	7933.08	0.07	340.64	30.99
AD18	71	Male	7	4	793.52	6088.05	0.13	753.72	172.75
AD19	67	Female	7	5	436.76	11101.43	0.04	310.89	51.09
AD20	58	Male	8	6	707.42	8630.71	0.08	546.35	86.88
AD21	57	Female	10	7	557.88	12600.88	0.04	740.81	89.07
AD22	48	Female	12	2	199.62	2222.11	0.09	229.12	35.21
AD23	55	Female	13	1	435.98	7192.9	0.06	932.16	265.97
AD24	52	Male	13	6	544.48	9071.14	0.06	893.61	126.78
AD25	53	Male	15	3	331.28	8775.14	0.04	1096.58	137.15
AD26	73	Female	15	1	287.88	2277.05	0.13	542.98	152.52
AD27	69	Female	16	1	358.8	9708.86	0.04	1054.26	130.39
AD28	70	Female	21	2	483.86	13735.12	0.04	846.79	76.83
AD29	67	Female	9	4	413.86	12339.88	0.04	645.81	151.65
AD30	59	Female	0	11	375.18	7772.2	0.05	448.21	39.68

Units of $A\beta_{1-42}$, $A\beta_{1-40}$, p-Tau, and t-Tau: pg/ml

Table S3: Information of normal individuals

	Age	Gender	MoCA	HAMD
CON1	82	Male	28	2
CON2	57	Male	30	3
CON3	61	Female	30	3
CON4	57	Male	30	3
CON5	70	Male	29	2
CON6	54	Female	30	4
CON7	72	Male	29	1
CON8	75	Male	28	1
CON9	67	Male	26	1
CON10	43	Male	30	0
CON11	62	Male	30	1
CON12	59	Female	30	0
CON13	77	Female	29	1
CON14	69	Male	30	2
CON15	64	Female	30	0
CON16	70	Female	28	1
CON17	48	Male	29	2

Table S4: Dual luciferase 3′UTR sequences

	3′UTR sequences
TLR4 mmu-miR-451a wild type	5′-GAAAGGAGAACCAGTCTTCACT<br>GGGCCTTTTGAATACAAGCCATGT<br>CATGTTCTGTGTTTCAGTTGCTTT<br>AGAAGAGTATTGATAGTTTCAACT<br>GAACTGAACGGTTTCTTACTTTCC<br>CTTTTTTCTACTGAATGCAATATT<br>AAATAGCTCTTTTTGAGAGGTCTT<br>CATTCCAATTTCATCTTCCATTTTA<br>TGTCATTTTCTTTTCT-3′
TLR4 mmu-miR-451a mutant	5′-GAAAGGAGAACCAGTCTTCACT<br>GGGCCTTTTGAATACAAGCCATGT<br>CATGTTCTGTGTTTCAGTTGCTTT<br>AGAAGAGTATTGATAGTTTCAACT<br>GAACTGAAGCGAATCTTACTTTCC<br>CTTTTTTCTACTGAATGCAATATT<br>AAATAGCTCTTTTTGAGAGGTCTT<br>CATTCCAATTTCATCTTCCATTTTA<br>TGTCATTTTCTTTTCT-3′

Table S5: Antibodies information

	Source	Catalog number	Host species	Dilution
NeuN	Millipore	MABN140	Rabbit	IF 1:500
IBA1	WAKO	019-19741	Rabbit	IF 1:600
6E10	Biolegend	803001	Mouse	WB 1:1000<br>IF 1:500
APP	SIGMA	SAB4300464	Rabbit	WB 1:1000
PS1	SIGMA	PRS4203	Rabbit	WB 1:1000
LRP-1	Abcam	ab92544	Rabbit	WB 1:1000
NEP	Millipore	AB5458	Rabbit	WB 1:800
IDE	Abcam	ab32216	Rabbit	WB 1:1000
BACE1	Proteintech	12807-1-AP	Rabbit	WB 1:1000<br>IF 1:100
ADAM10	Millipore	AB19026	Rabbit	WB 1:1000
GAPDH	Proteintech	60004-1-Ig	Mouse	WB 1:2000
TLR4	ABclonal	A17436	Rabbit	WB 1:1000
P-IKKβ	CST	2697s	Rabbit	WB 1:1000
IKKβ	CST	8943S	Rabbit	WB 1:1000
p-P65	CST	3033s	Rabbit	WB 1:1000
P65	CST	8242	Rabbit	WB 1:1000
PSD95	Abcam	Ab18258	Rabbit	WB 1:1000
SYP	Proteintech	17785-1-AP	Rabbit	WB 1:1000
NLRP3	AdipoGen	AG-20B-0014-C100	Mouse	IF 1:200<br>WB 1:1000
GFAP	Abcam	Ab4674	Chicken	IF 1:500
sAPPβ	biolegend	813401	Rabbit	WB 1:1000
MAP2	millipore	Ab5622	Rabbit	IF 1:200
Caspase-1	SANTA	SC-56036	Mouse	WB 1:1000
ASC	SANTA	SC-271054	Mouse	WB 1:1000

Table S6: Primer information

	Forward primer	Reverse primer
miR-30a-5p	CGCGGAAGGTCAGCTCCTAC	AGTGCAGGGTCCGAGGTATT
miR-345	GCTGACCCCTAGTCCAGTGCTT	Universal PCR Primer R
miR-375	AAGCTTTGTTCGTTCGGCTC	GTATCCAGTGCGAATACCTC
miR-451a	GCGAAACCGTTACCATTACTGAGTT	Universal PCR Primer R
miR-4726-3p	AGAGGAGCCTGGAGTGGTC	CGGCCAGAGGGAACCTG
miR-765	TGGAGGAGAAGGAAGGTG	GAACATGTCTGCGTATCTC
miR-1257	AGTGAATGATGGGTTCTGACCAAA	Universal PCR Primer R
miR-486-5p	CTCGCTTCGGCAGCACA	ACGCTTCACGAATTTGCGT
miR-320e	AGTGCGAACTGTGGCGAT	ATAACATTCAACGCTGTCGGTGA
U6	Universal U6 Primer F	CACGAATTTGCGTGTCATCCTT
ATF2	CCGTTGCTATTCCTGCATCAA	TTGCTTCTGACTGGACTGGTT
FLI1	CAGTTACCTCAGGGAAAACCC	TGCTCAGTGTTCTTGCCCAT
MYC	GCTCTGCTCTCCGTCCTATGT	CAGTCCTGGATGATGATGTTCTTGA
NFIB	TAACGGCAGTGGTCAAGT	TGCTCAGGGTCACAGGTC
PPARGC1A	TATGGAGTGACATAGAGTGTGCT	CCACTTCAATCCACCCAGAAAG
Six1	ATGCTGCCGTCGTTTGGTT	CCTTGAGCACGCTCTCGTT
Tbx19	TCTCGCCTGCTTAACGTGG	CCAGCCCTGTGACACTAATCTT
TLR4	ATGGCATGGCTTACACCACC	GAGGCCAATTTTGTCTCCACA
WWTR1	CATGGCGGAAAAAGATCCTCC	GTCGGTCACGTCATAGGACTG
YBX1	CAGACCGTAACCATTATAGACGC	ATCCCTCGTTCTTTTCCCCAC
Zic3	TGCTGCCAGTTCAGGCTATG	GCAGAAGGGGTTTTAGTGGTATC
GAPDH	AGGTCGGTGTGAACGGATTTG	TGTAGACCATGTAGTTGAGGTCA

Table S7: Involvements of miRNAs in neurological diseases

miRNAs	Changes in diseases	Reference and database
miR-30a-5p	Up-regulated in serum from AD patients	[59]
	Up-regulated in CSF from EO-FAD patients	[81]
	Up-regulated in serum from post-stroke depression patients	[82]
miR-345	Up-regulated in serum from major depression patients	[83]
	Up-regulated in serum from C9orf72-associated frontotemporal dementia patients	[84]
	Up-regulated in CSF from EO-FAD patients	[81]
miR-375	Up-regulated in CSF from AD patients	[85]
	Up-regulated in CSF and serum from major depression patients	[34]
miR-451a	Up-regulated in serum-EXs from multiple sclerosis patients	[86]
	Up-regulated in plasma from vascular dementia patients	[87]
	Down-regulated in CSF-EXs from AD patients	[60]
	Down-regulated in amyotrophic lateral sclerosis patients	[88]
miR-4726-3p	Up-regulated in serum from major depression patients	[88]
	Up-regulated in temporal cortex from AD patients	GSE157239
miR-765	Up-regulated in serum from major depression patients	[88]
	Down-regulated in temporal cortex from AD patients	GSE157239
miR-486-5p	Up-regulated in temporal cortex from AD patients	GSE157239
	Down-regulated in serum from depressed patients	[89]
	Down-regulated in CSF from AD patients	[90]
miR-320e	Down-regulated in serum from major depression patients	[88]
	Up-regulated in temporal cortex from AD patients	GSE157239

Supplementary figure and Legends

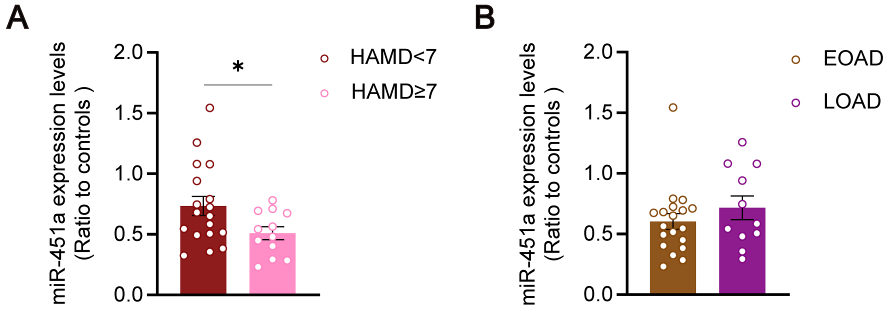

Figure S1. CSF miR-451a levels in different types of AD patients. (A) miR-451a expression levels in the AD patients with depression (HAMD ≥ 7) and without depression (HAMD < 7). **(B)** miR-451a expression levels in the EOAD patients (age < 65) and LOAD patients (age ≥ 65). Data are presented as mean ± SEM. n = 30 for AD patients (12 for HAMD ≥ 7 and 18 for HAMD < 7; 19 for EOAD and 11 for LOAD). Significance was evaluated with Student's *t*-test. *$p < 0.05$.

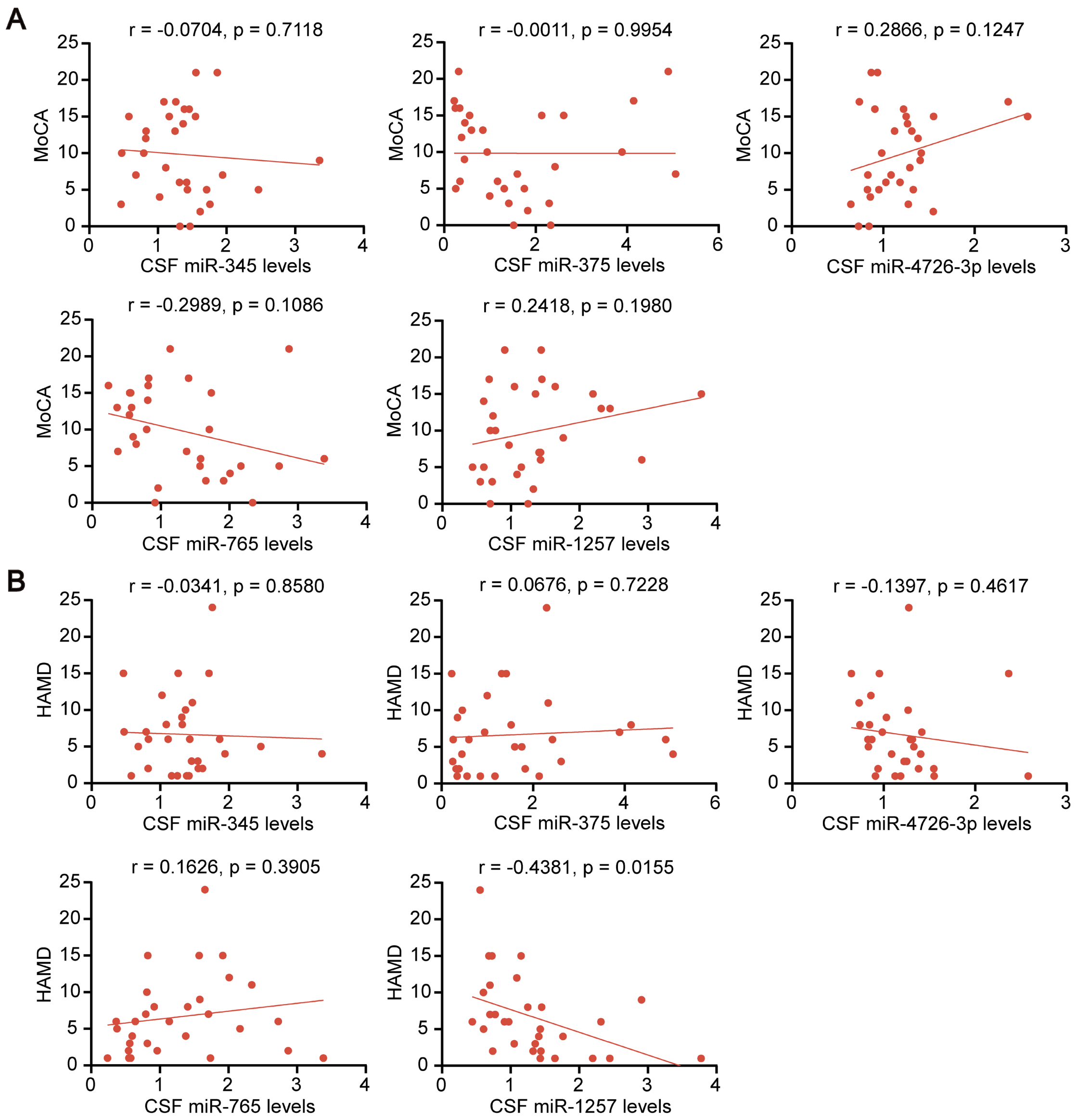

Figure S2. The correlation analysis of CSF miRNA levels, HAMD, and MoCA score of AD patients. (A) The correlation analysis of MoCA scores and the relative levels of miR-345, miR-375, miR-4726-3p, miR-765, and miR-1257 in our enrolled AD patients, respectively. **(B)** The correlation analysis of HAMD scores and the relative levels of each of the miRNAs mentioned above. n = 30 per group. Significance was evaluated with Pearson's correlation test.

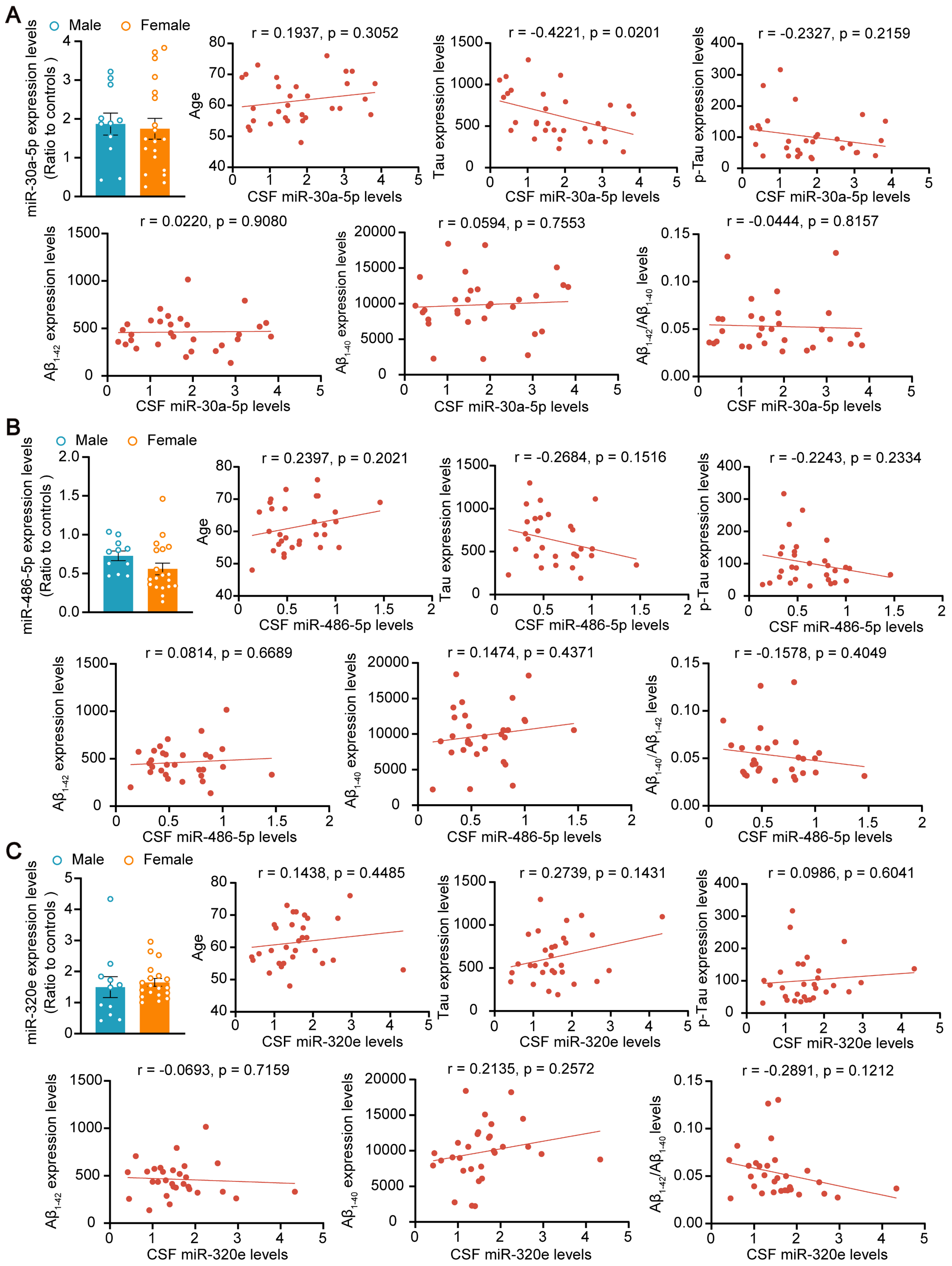

Figure S3. The analysis of relationship between CSF miRNA levels and indicators of AD patients. **(A-C)** Analysis of the relationship between gender, age, Aβ levels, and Tau levels of AD patients and miR-30a-5p, miR-486-5p, and miR-320e levels in the CSF. n = 30 for AD

patients (11 males and 19 females). Significance was evaluated with Student's *t*-test for gender analysis and Pearson's correlation test for other analyses.

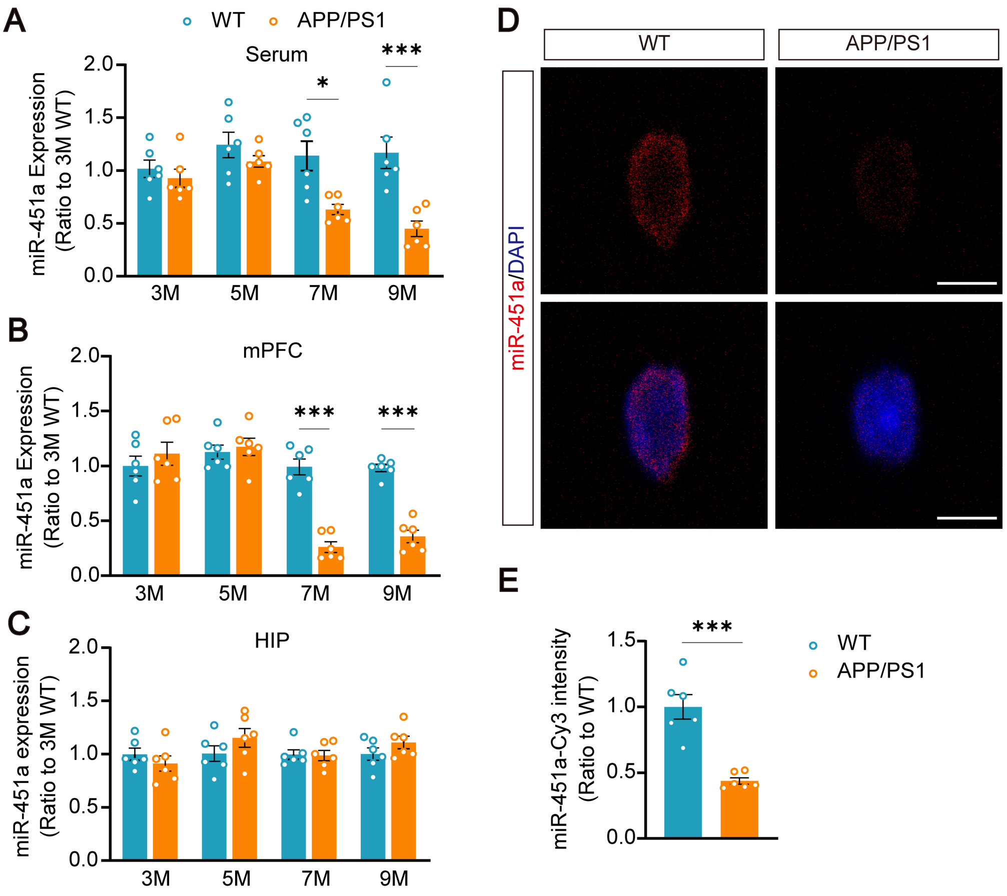

Figure S4. Changes of miR-451a in the serum, mPFC, and CSF of APP/PS1 mice. (A-C) Relative expression of miR-451a in the serum **(A)**, mPFC **(B),** and hippocampus **(C)** of APP/PS1 mice and WT mice at different months. **(D)** Representative images of miR-451a in CSF smear at 7 months of APP/PS1 mice and WT mice. Scale bar, 5 µm. **(E)** The quantification of fluorescence intensity for miR-451a in the CSF smear samples. Data are presented as mean ± SEM. n = 6 per group.

Significance was evaluated with two-way ANOVA with Tukey post-hoc test (**A-C**) or Student's *t*-test **(E)**. $*p < 0.05$, $***p < 0.001$.

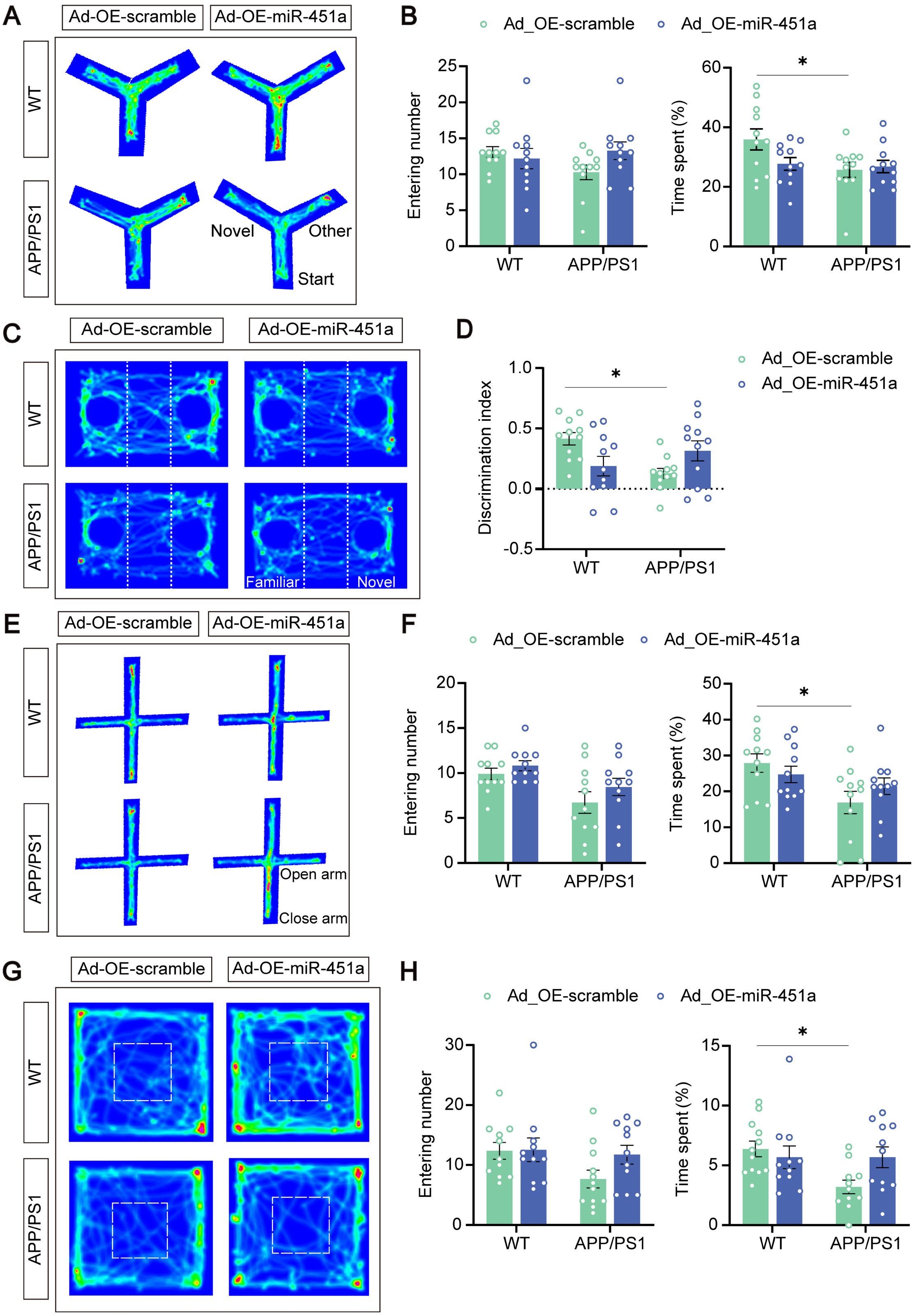

Figure S5. Overexpression of miR-451a did not rescue short-term

memory and anxiety-like behavior in APP/PS1 mice. (**A**) Movement tracing in the Y-maze. (**B**) Quantification of entering number and percentage of time spent in the novel arm. (**C**) Movement tracing during the novel object recognition. (**D**) Discrimination index in the novel object recognition. (**E**) Movement tracing during the elevated plus maze test. (**F**) The entering number and percentage of time spent in the open arm. (**G**) Movement tracing during the open field test. (**H**) The entering number and percentage of time spent in the center area. Data are presented as mean ± SEM. n = 11 per group. Significance was evaluated with two-way ANOVA with Tukey post-hoc test. *$p < 0.05$.

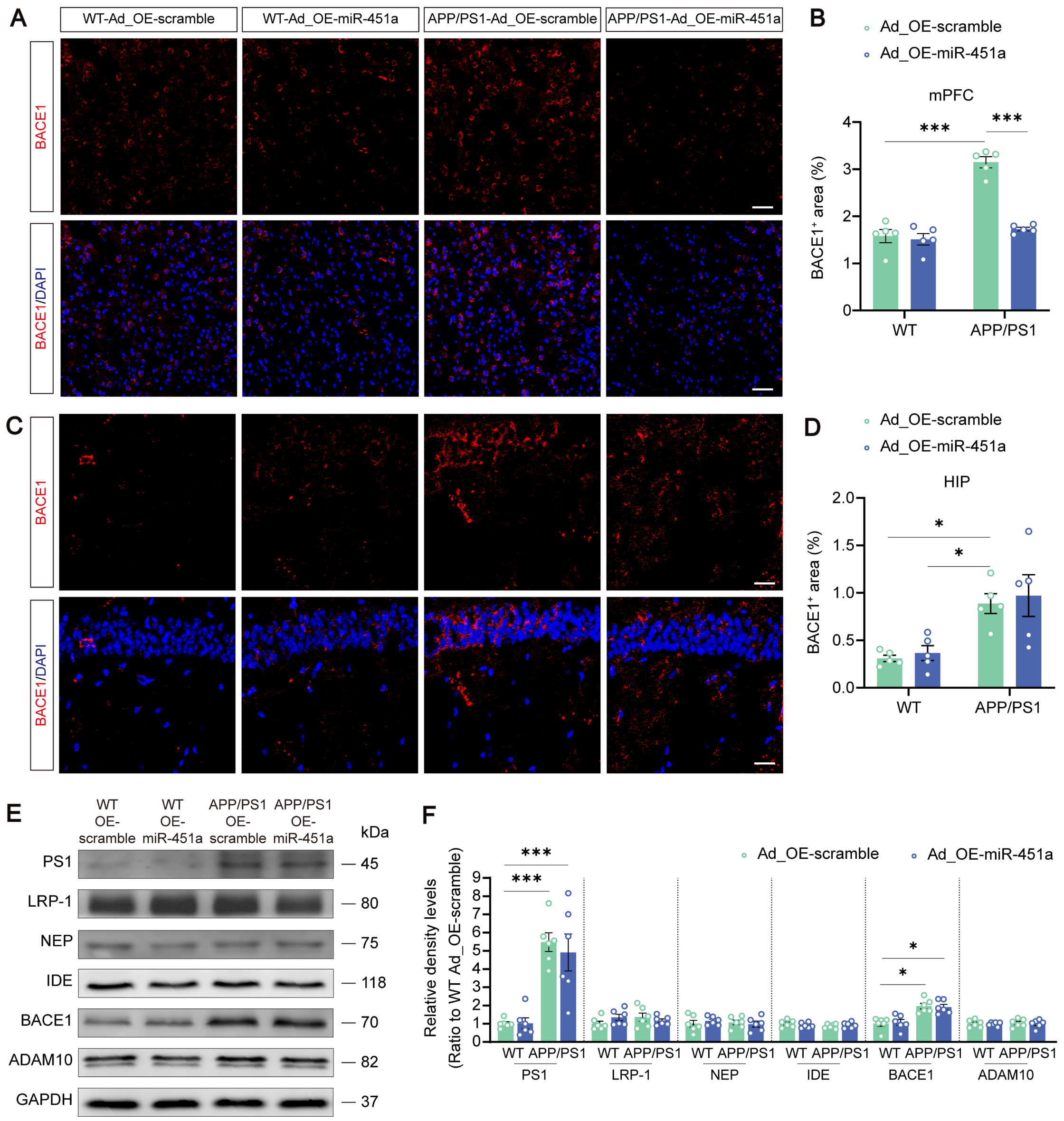

Figure S6. Overexpression of miR-451a decreased BACE1 expression in the mPFC but not in the hippocampus. (A) Representative images of BACE1 immunofluorescence in the mPFC of APP/PS1 mice and WT mice with an injection of Ad_OE-miR-451a or Ad_OE-scramble in the mPFC. Scale bar, 50 μm. **(B)** The percentage of BACE1$^+$ area in the mPFC. **(C)** Representative images of BACE1 immunofluorescence in the hippocampus (HIP) of APP/PS1 mice and WT mice with an injection of Ad_OE-miR-451a or Ad_OE-scramble within the mPFC. Scale bar, 50

μm. **(D)** The percentage of BACE1^{+} area in the hippocampus. **(E, F)** Representative Western blot bands and densitometry analysis of PS1, LRP-1, NEP, IDE, BACE1, and ADAM10 in the hippocampus of each group. Data are presented as mean ± SEM. n = 5 per group for **(B, D)** and n = 6 per group for **(F)**. Significance was evaluated with two-way ANOVA with Tukey post-hoc test. $*p < 0.05$, $***p < 0.001$.

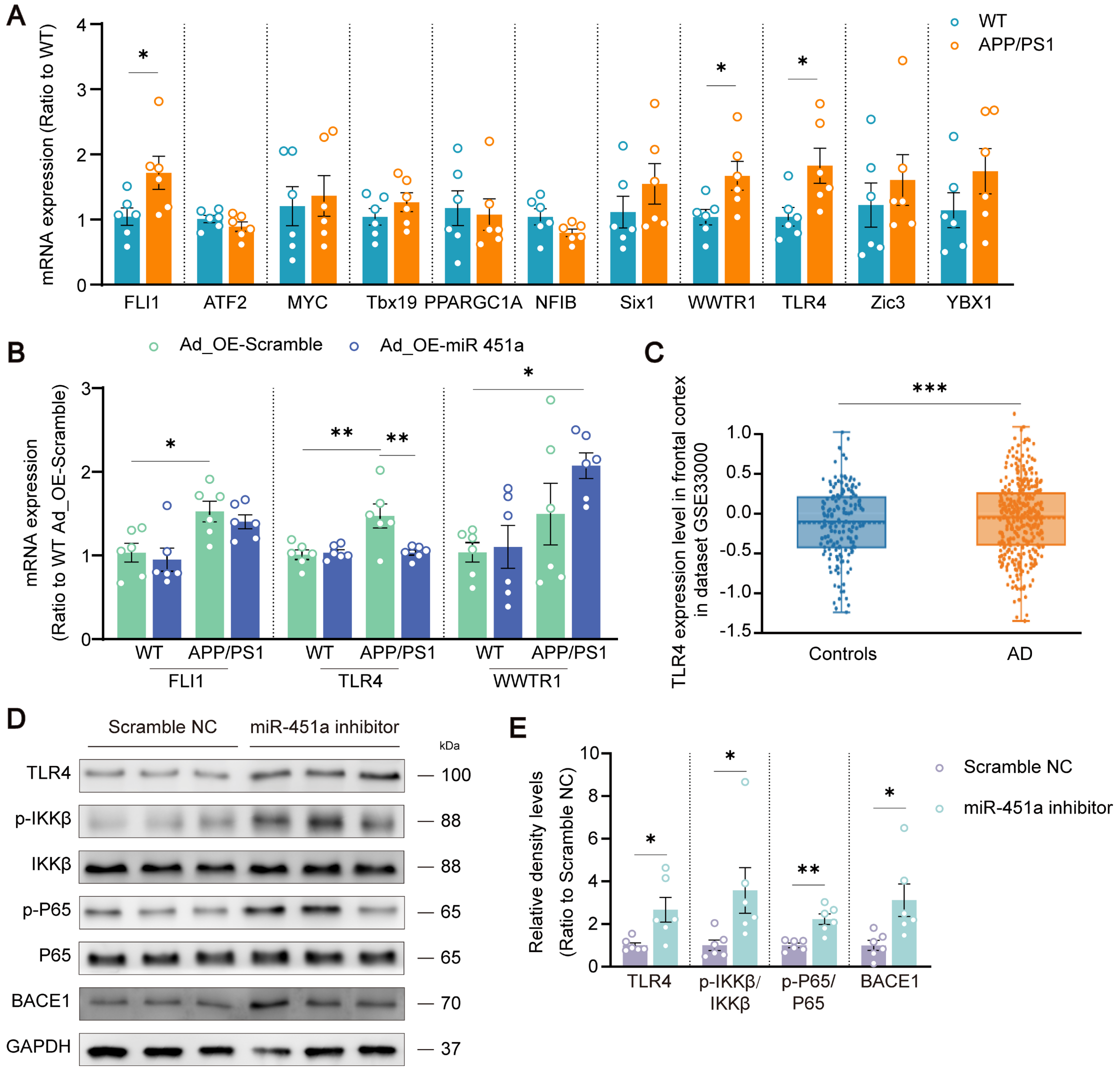

Figure S7. MiR-451a inhibited TLR4/IKKβ/NF-κB signal pathway. **(A)** qRT-PCR verified the 11 candidate genes on the mPFC samples from APP/PS1 and WT mice. **(B)** qRT-PCR examined the 3 verified candidates:

FLI1, TLR4, and WWTR1 on the mPFC samples from APP/PS1 and WT mice injected AAV-OE-scramble and AAV-OE-miR-451a. **(C)** The expression level of TLR4 from the dataset GSE33000 of AD patients and controls. **(D, E)** Representative Western blot bands and densitometry analysis of TLR4, p-IKKβ/IKKβ, and p-NF-κB/NF-κB in the N2a cells after treatment of miR-451a inhibitor. Data are presented as mean ± SEM. n = 6 per group for **(A, B, E)** and n = 624 per group for **(C)**. Significance was evaluated with Student's *t*-test **(A, C, E)** or two-way ANOVA with Tukey post-hoc test **(B)**. *$p < 0.05$, **$p < 0.01$, ***$p < 0.001$.

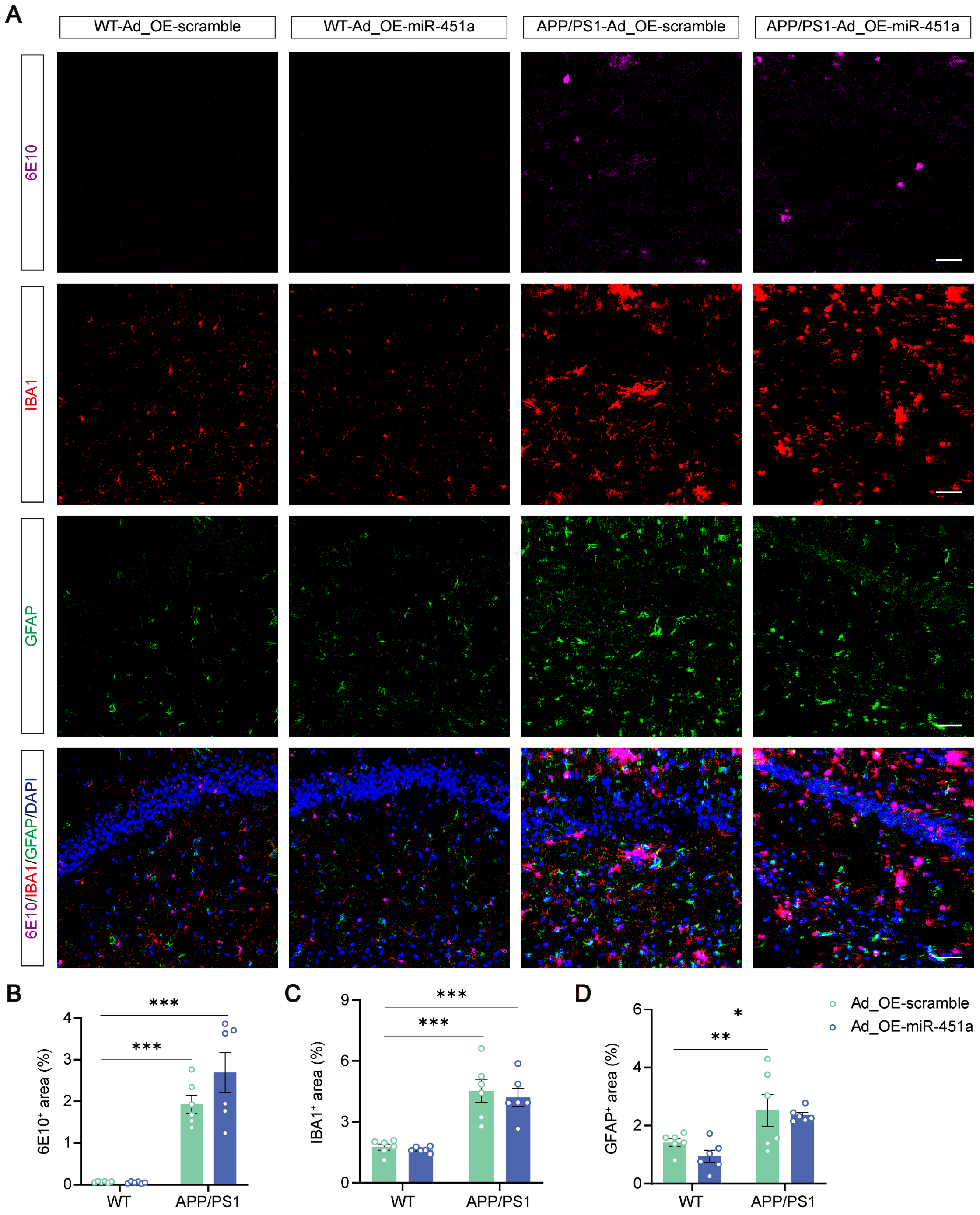

Figure S8. Overexpression of miR-451a did not inhibit neuroinflammation in the hippocampus of APP/PS1 mice. (A) Representative image of 6E10 (Magenta), IBA1 (Red), GFAP (Green), and DAPI (Blue) in the hippocampus of WT mice and APP/PS1 mice with an injection of Ad_OE-miR-451a or Ad_OE-scramble within the

mPFC. Scale bar, 50 μm. (**B**) The percentage area positive for 6E10. **(C)** The percentage area positive for IBA1. **(D)** The percentage area positive for GFAP. Data are presented as mean ± SEM. n = 6 per group. Significance was evaluated with two-way ANOVA with Tukey post-hoc test. *$p < 0.05$, **$p < 0.01$, ***$p < 0.001$.

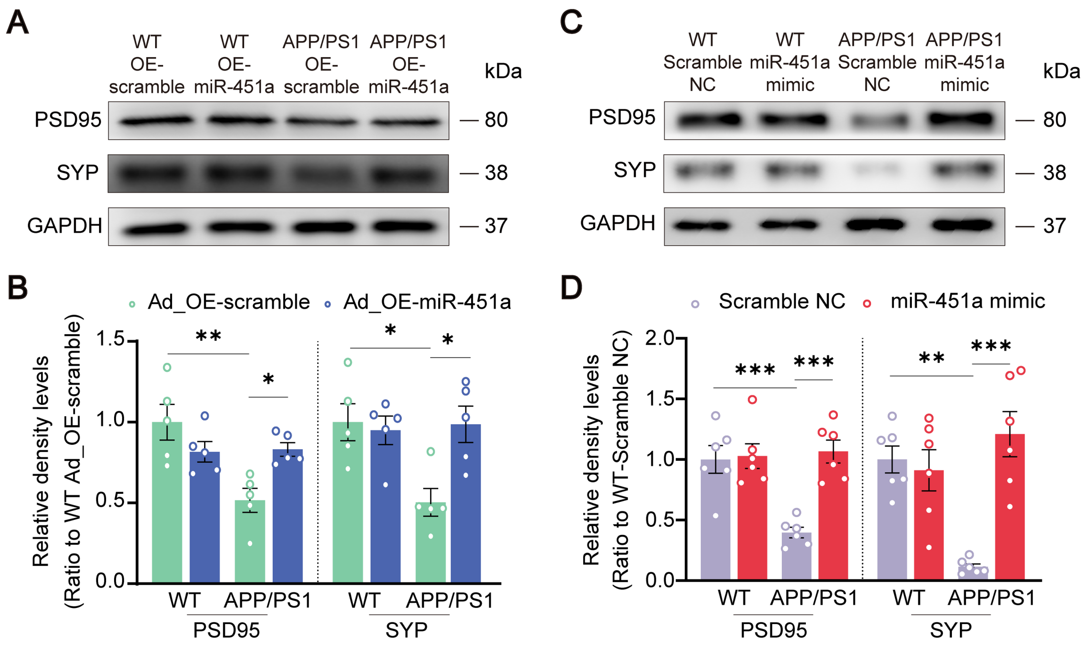

Figure S9. MiR-451a improved synaptic protein expression in APP/PS1 mice. (A, B) Representative Western blot bands and densitometry analysis of PSD95 and SYP in the mPFC of WT mice and APP/PS1 mice injected Ad_OE-miR-451a or Ad_OE-scramble. **(C, D)** Representative Western blot bands and densitometry analysis of PSD95 and SYP in the primary neurons after treatment of miR-451a mimic. Data are presented as mean ± SEM. n = 5 or 6 per group. Significance was evaluated with two-way ANOVA with Tukey post-hoc test. *$p < 0.05$, **$p < 0.01$, ***$p < 0.001$.

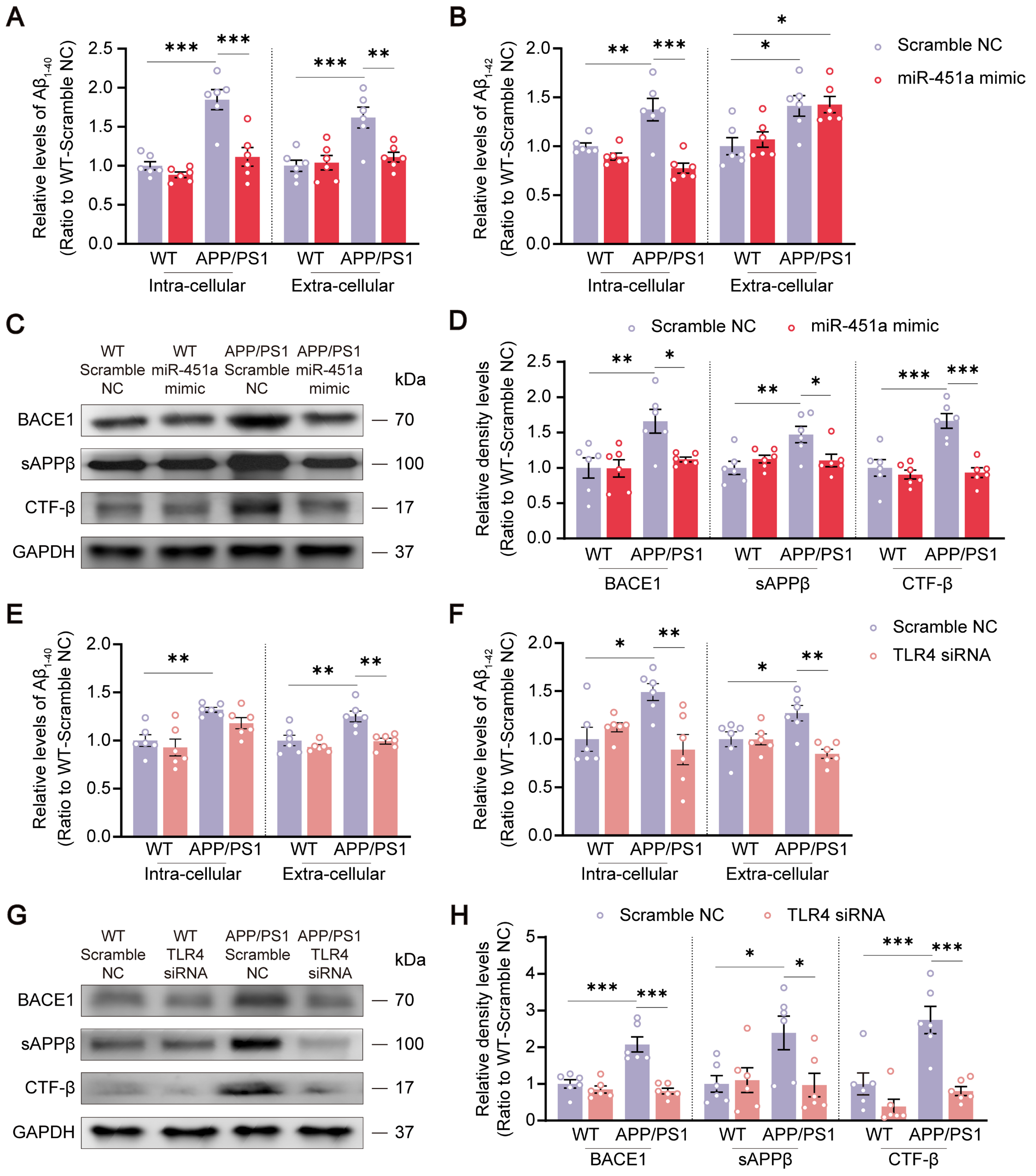

Figure S10. TLR4 mediated the inhibitory effect of miR-451a on BACE1 expression in primary cortex neurons from APP/PS1 mice. (A, B) ELISA analyses of Aβ1-40 levels **(A)** and Aβ1-42 levels **(B)** in the primary neurons (Intra-cellular) and its supernatant (Extra-cellular) treated with either miR-451a mimic (50 nM) or scrambled control. (**C, D)** Representative bands of Western blot and densitometry analysis of BACE1, sAPPβ, and CTF-β in primary neurons treated with either

miR-451a mimic (50 nM) or scrambled controls. **(E, F)** ELISA analyses of Aβ_{1-40} levels **(E)** and Aβ_{1-42} levels **(F)** in the primary neurons (Intra-cellular) and its supernatant (Extra-cellular) treated with either TLR4 siRNA (100 nM) or scrambled control. **(G, H)** Representative bands of Western blot and densitometry analysis of BACE1, sAPPβ, and CTF-β in primary neurons treated with either TLR4 siRNA (100 nM) or scrambled controls. Data are presented as mean ± SEM. n = 6 per group. Significance was evaluated with two-way ANOVA with Tukey post-hoc test. *$p < 0.05$, **$p < 0.01$, ***$p < 0.001$.
